# Differential associations of plasma lipids with incident dementia and dementia subtypes in the 3C Study: A longitudinal, population-based prospective cohort study

**DOI:** 10.1371/journal.pmed.1002265

**Published:** 2017-03-28

**Authors:** Sabrina Schilling, Christophe Tzourio, Aïcha Soumaré, Sara Kaffashian, Jean-François Dartigues, Marie-Laure Ancelin, Cécilia Samieri, Carole Dufouil, Stéphanie Debette

**Affiliations:** 1 University of Bordeaux, Bordeaux, France; 2 Inserm U1219, Bordeaux, France; 3 Department of Public Health, Bordeaux University Hospital, Bordeaux, France; 4 ISPED, University of Bordeaux, Bordeaux, France; 5 Inserm U1061, Montpellier, France; 6 University of Montpellier, Montpellier, France; 7 Memory Clinic, Department of Neurology, Bordeaux University Hospital, Bordeaux, France; 8 Framingham Heart Study, Department of Neurology, Boston University School of Medicine, Boston, Massachusetts, United States of America; University of California San Francisco Memory and Aging Center, UNITED STATES

## Abstract

**Background:**

Vascular risk factors have been proposed as important targets for the prevention of dementia. As lipid fractions represent easily modifiable targets, we examined the longitudinal relationship of baseline lipid fractions with 13-y incident dementia and its subtypes (Alzheimer disease [AD] and mixed or vascular dementia) in older community-dwelling persons.

**Methods and findings:**

Non-institutionalized persons aged 65+ y (*n =* 9,294) were recruited for the Three-City Study (3C Study), a population-based cohort study from the electoral rolls of the cities of Dijon, Bordeaux, and Montpellier, France, between March 1999 and March 2001. Follow-up examinations were performed every 2 y after the baseline assessment. The final study sample comprised 7,470 participants from the 3C Study (mean age ± standard deviation [SD] 73.8 ± 5.3 y, 61.0% women) who were prospectively followed up for up to 13 y. Fasting lipid fractions (triglycerides [TGs], high-density lipoprotein cholesterol [HDL-C], low-density lipoprotein cholesterol [LDL-C], total cholesterol [TC]) were studied as continuous variables, and results are reported per SD increase of each lipid fraction. Incident dementia and its subtypes were studied as censored variables using Cox models with age as time scale. Analyses were adjusted for sex, study center, and educational level, as well as vascular risk factors and *apolipoprotein E* (*APOE*) ε4 genotype. We corrected for multiple testing, yielding a significance threshold of 0.0169. *p*-Values above the significance threshold but less than 0.05 were considered nominally significant.

During a mean (± SD) follow-up period of 7.9 ± 3.6 y, 779 participants developed incident dementia (*n =* 532 AD and *n =* 154 mixed or vascular dementia). Higher LDL-C and TC concentrations at baseline were associated with an increased risk of AD (hazard ratio [HR] per SD increase = 1.13 [95% CI 1.04–1.22], *p =* 0.0045, and HR = 1.12 [1.03–1.22], *p =* 0.0072, respectively). These associations were largely unchanged after adjustment for vascular risk factors and were attenuated after adjustment for *APOE*ε4 (HR per SD increase = 1.12 [1.03–1.23], *p =* 0.0110, and HR = 1.12 [1.02–1.23], *p =* 0.0171, respectively). Higher TG concentrations at baseline were associated with an increased risk of all dementia (HR per SD increase = 1.11 [1.03–1.19], *p =* 0.0044) and mixed or vascular dementia (HR = 1.21 [1.04–1.41], *p =* 0.0163). However, these associations disappeared after adjusting for vascular risk factors (HR = 1.07 [0.98–1.17], *p =* 0.1374, and HR = 1.17 [0.96–1.42], *p =* 0.1206, respectively). Main limitations of the study include interval censoring of incident dementia cases, potential selective survival bias, and the fact that variation in lipid concentrations during follow-up could not be accounted for in the analyses.

**Conclusions:**

In a large population-based sample of older community-dwelling persons with up to 13 y of follow-up, we observed that higher LDL-C and TC concentrations were associated with an increased risk of AD. This result was independent of vascular risk factors and was attenuated after adjustment for *APOE*ε4 carrier status. TG and HDL-C concentrations were not associated with risk of incident dementia or its subtypes after accounting for vascular risk factors.

## Abstract 2

In a longitudinal population-based prospective cohort study, Stéphanie Debette and colleagues investigate differential associations of plasma lipids with dementia and dementia subtypes.

## Author summary

**Why was this study done?:**

- It is not currently possible to prevent or treat dementia, the frequency of which is increasing dramatically with aging populations worldwide.
- As lipid concentrations represent easily modifiable potential targets for prevention, exploring their relation with dementia risk is of major interest.

**What did the researchers do and find?:**

- We explored whether there was an association between lipid concentrations measured in 7,470 dementia-free individuals from the general population aged 65 years or older and the occurrence of dementia after up to 13 years of follow-up.
- We studied the occurrence of dementia overall and of its two main subtypes, Alzheimer disease and mixed or vascular dementia.
- Dementia occurred in 779 individuals during follow-up, and we found that higher total cholesterol and higher low-density lipoprotein cholesterol concentrations at baseline were associated with an increased risk of Alzheimer disease, but these associations were attenuated after adjustment for *APOE*ε4, a genetic risk factor for Alzheimer disease.
- We did not observe any robust association between higher triglycerides or high-density lipoprotein cholesterol concentrations and risk of dementia.

**What do these findings mean?:**

- If our findings are confirmed in independent studies, it would mean that it might be useful to explore the potential benefit of using lipid-lowering drugs to prevent dementia in older community-dwelling persons.

## Introduction

Dementia refers to a group of neurological disorders characterized by memory loss, cognitive impairment, and disability in activities of daily living. As the primary risk factor for dementia is old age, the prevalence of dementia is increasing dramatically with aging populations worldwide. As no effective preventive treatment is currently available, the societal burden of dementia is huge and threatening to increase further [1]. The most common form is Alzheimer disease (AD), a neurodegenerative disease representing 50%–70% of dementia cases. Cerebrovascular disease is also a major contributor to dementia risk, often in conjunction with neurodegenerative lesions [2]. Vascular risk factors have been proposed as important targets for the prevention of dementia, with around a third of AD cases being attributable to potentially modifiable risk factors, especially vascular risk factors [3,4], although trials have been inconclusive so far. As lipid fractions represent easily modifiable potential targets for prevention, exploring their relation with dementia risk is of major interest. So far, published studies have shown inconsistent results, including associations of adverse lipid profiles with an increased dementia risk [5–10], absence of an association [11–17], or even inverse associations [18–20]. Important differences between studies—regarding the timing of the measurement of lipid fraction in relation to the diagnosis of dementia, the age at which plasma lipid concentrations were measured, and the duration of follow-up—might at least partly explain these discrepancies. Significant associations of high cholesterol concentrations with dementia [6], AD [7–10], or dementia death [21,22] are described mostly in studies where lipid concentrations were measured in midlife and/or participants were followed for a long period until advanced late life, hence with a long exposure to high cholesterol concentrations. In contrast, studies with lipid measurements in later life or short follow-up periods not reaching the ages at which dementia prevalence is highest either do not observe any association [4,13–15] or sometimes observe inverse relations with dementia risk [18,19]. Moreover, most studies have focused either on total cholesterol (TC) and low-density lipoprotein cholesterol (LDL-C) or on triglycerides (TGs) and high-density lipoprotein cholesterol (HDL-C); few studies have studied all fractions simultaneously in the same dataset. Interestingly, we and others have recently shown that lipid fractions, notably higher TG concentrations, are significantly associated with white matter hyperintensity (WMH) volume on brain MRI [23–25], a powerful predictor of dementia risk [26]. In the present work, we aimed to evaluate the relationship of lipid fractions (TGs, HDL-C, LDL-C, and TC) with incident dementia in a large cohort of community-dwelling older individuals over a follow-up period of 13 y.

## Methods

### Ethics statement

The Ethics Committee of Kremlin-Bicêtre University Hospital approved study protocols, and each participant signed a written informed consent.

### Study population

The Three-City Study (3C Study) is a longitudinal, population-based prospective cohort study, described in detail elsewhere [27]. Briefly, 9,294 non-institutionalized persons aged 65+ y were recruited from the electoral rolls of Dijon, Bordeaux, and Montpellier, France, between March 1999 and March 2001. Extensive follow-up examinations were performed every 2 y after the baseline assessment, comprising standardized questionnaires, clinical examinations, and detailed cognitive assessment. The third follow-up examination consisted of a self-questionnaire or a phone interview for participants who had refused to or could not fill in the questionnaire. We excluded participants with brain tumor (*n =* 8), prevalent dementia (*n =* 214), or missing data for either lipid concentrations (*n =* 556) or educational level (*n =* 5). We also removed participants with Mini-Mental State Examination (MMSE) score less than 24 at baseline (*n =* 432) or with missing data for MMSE (*n =* 40), as these individuals might have had an undiagnosed dementia, with lipid concentrations already impacted by metabolic changes secondary to the disease [28], thus leading to a sample of 8,039 participants at baseline. Incident dementia cases were prospectively ascertained over a 13-y follow-up period. Follow-up data on the outcome of interest were available in 7,470 of the 8,039 participants (>92.9%), comprising our final study sample (see S1 Fig).

### Outcome ascertainment and definition of variables

#### Dementia ascertainment

Dementia status was evaluated prospectively by an expert panel. In Bordeaux and Montpellier, all participants were examined by a neurologist. In Dijon, due to the large number of participants, a two-step procedure was used [27]: (i) a careful neuropsychological evaluation carried out by a trained psychologist and (ii) an examination by a neurologist for those who screened positive at step 1 based on MMSE and Isaacs’ Set Test, a measure of verbal fluency and response rapidity that consists of generating words belonging to given semantic categories (e.g., animal names) in 15 s [27]. Isaacs’ Set Test has been reported to show the earliest decline in the decade preceding dementia diagnosis [29,30]. Cutoff scores were defined according to educational level as previously described [31]. Finally, in all centers, the diagnosing examination and subtype classification of all suspected prevalent and incident dementia cases were performed by an independent committee of neurologists following DSM-IV criteria [27,32]. The final diagnosis of dementia and subtype was made based on all available information, including data on cognitive functioning and daily activities, severity of cognitive disorders (Clinical Dementia Rating Scale), and, where possible, hospitalization records, CT scans (which were most often used at the beginning of the follow-up period) and magnetic resonance images [27], and functional assessment, which included assessment of disabilities using the Katz Index of Activities of Daily Living [33], the Lawton Instrumental Activities of Daily Living Scale [34], and the Rosow and Breslau scales [27,35]. Dementia subtypes included AD, vascular dementia, and mixed dementia. Due to small numbers in the last two categories, these were pooled for analyses. Dementia subtyping was based, for AD, on National Institute of Neurological and Communicative Disorders and Stroke–Alzheimer’s Disease and Related Disorders Association criteria, and, for vascular dementia, on National Institute of Neurological Disorders and Stroke–Association Internationale pour la Recherche et l’Enseignement en Neurosciences criteria [36,37]. Mixed dementia was defined as diagnosis of AD with either cerebrovascular lesions on brain imaging or a documented history of stroke and presence of prominent executive function deficits in addition to an AD-type cognitive profile.

#### Laboratory testing

Centralized measurements of baseline fasting serum TC, HDL-C, and TG were performed using enzymatic methods. LDL-C was calculated with the Friedewald formula [38] (LDL-C = TC − HDL-C − [TGs/2.2]) and was considered missing for TG values > 400 mg/dl (4.52 mmol/l).

#### Covariates

The following covariates were measured at baseline. Hypertension was defined by systolic blood pressure ≥ 140 mm Hg or diastolic blood pressure ≥ 90 mm Hg or antihypertensive drug intake. Body mass index (BMI) was calculated as the ratio of weight (in kilograms) to the square of height (in meters). Diabetes was defined as fasting blood glucose ≥ 7 mmol/l or antidiabetic drug intake or medical history of diabetes. Hypercholesterolemia was defined as fasting TC ≥ 6.2 mmol/l or lipid-lowering drug intake (fibrate, statin, or bile acid sequestrant). Smoking status was categorized as never, former, or current smoker. History of cardiovascular disease was defined as a history of stroke, myocardial infarction, angina pectoris, or peripheral artery disease. Methods for genotyping the *APOE* epsilon polymorphism have been described previously [39]. *APOE*ε4 carrier was defined as the presence of at least one ε4 allele. Educational level at baseline was defined as a six-class variable (no education, primary school, secondary school with certificate of vocational aptitude, secondary school with secondary education certificate, baccalaureate or equivalent, university or equivalent).

### Statistical analyses

To study the association between baseline lipid concentrations and 13-y incident dementia, we used Cox models, using age as time scale and birth as time origin. This allowed us to avoid the non-proportionality of dementia risk with age [40]. Baseline age was the age at which participants entered the cohort. Data were censored at the age of dementia diagnosis for cases (median of the interval of the last follow-up visit without dementia and the first follow-up visit with dementia) or at age at last follow-up for controls. This model also accounted for left truncation and corrected for the bias introduced by including at baseline only individuals who did not develop dementia before inclusion. We verified the proportional hazard assumption using proportionality tests that assess the statistical significance of interaction terms between time (age at last follow-up or dementia occurrence) and the variables in the model. Since this assumption was not verified for educational level and antihypertensive drug intake, all Cox models also included an interaction term for these covariates with time (the interaction with antihypertensive drug intake being present only in models adjusted for vascular risk factors). Analyses were initially adjusted for study center, sex, educational level, and interaction between educational level and time (Model 1). Then additional adjustments were made for (i) vascular risk factors: BMI, systolic blood pressure, anti-hypertensive drug intake, interaction between antihypertensive drug intake and time, smoking status, diabetes, lipid-lowering drug intake, other lipid concentrations (analyses on TC were adjusted for TGs), and history of cardiovascular disease (model 2) and (ii) vascular risk factors and *APOE*ε4 genotype (model 3). Lipid concentrations were studied as continuous variables, and results are reported per increase in standard deviation (SD) of each lipid fraction. Of note, TG concentrations were log-transformed for analyses to remove skewness. When examining dementia subtypes, all subtypes that were not the primary outcome of interest were censored at the age of their diagnosis. We corrected for multiple testing by accounting for the number of independent lipid phenotypes examined, based on a previously described method [41], yielding a significance threshold of 0.0169 (based on a family-wise error rate of 0.05 and three independent tests) that was applied to all analyses. *p*-Values greater than 0.0169 but less than 0.05 were considered nominally significant.

In secondary analyses, we first replaced overall lipid-lowering drug intake by statin intake when adjusting for vascular risk factors. Second, we ran sensitivity analyses censoring non-demented individuals at the age at last follow-up or age at death, instead of age at last follow-up only, in order to account for the competing risk of death. Third, we also performed Cox models using time-on-study as the time scale in order to generate cumulative incidence graphs adjusted for age, sex, and education and stratified on lipid concentration (top quartile [or bottom quartile for HDL-C] versus the rest). Fourth, we examined the robustness of our results by stratifying on *APOE*ε4 carrier status, lipid-lowering drug intake, sex, median age, or educational level (using a dichotomized variable coding no education/primary school versus the rest) and formally tested for interaction with these variables. Fifth, we examined in greater depth the impact of lipid-lowering drug intake on associations. As the effect of lipid-lowering drugs on AD was reported to vary by sex [42], we also ran the analyses adjusted for lipid-lowering drug intake stratified by sex. Also, to take into account not only lipid-lowering drug intake but also the effect of lipid-lowering drugs on lipid concentrations, we stratified analyses on a three-class variable crossing lipid-lowering drug intake and TC concentration at baseline (i.e., no lipid-lowering drug intake, lipid-lowering drug intake and TC concentration at baseline < 6.2 mmol/l, lipid-lowering drug intake and TC concentration at baseline ≥ 6.2 mmol/l). Sixth, we explored the relationship between sex-specific lipid quartiles and dementia risk, and linearity was assessed using the method based on Cox models as described above. The hazard ratio (HR) of each class of the lipid fraction divided in four classes, in each of which lipid concentrations were replaced by the value of the mean of the corresponding quartile minus that of the first quartile, was compared to the confidence interval of the HR of the corresponding quartile. The log-linearity hypothesis is acceptable if the HRs calculated for the classes are included in the confidence intervals from the corresponding quartile [43]. We also used plots of cumulative Martingale residuals against the continuous variable of interest (LDL-C, TC, or TG) [44].

Analyses were performed using SAS software version 9.3 (SAS Institute).

## Results

Our sample comprised 7,470 individuals, the characteristics of whom are described in Table 1. Compared to individuals who could not be included in the present study, individuals included were younger; were less likely to have hypercholesterolemia, hypertension, diabetes, and a history of cardiovascular disease; and were less likely to be *APOE*ε4 carriers, but were more likely to take lipid-lowering medication. Included individuals also had lower TG and higher HDL-C concentrations, lower systolic and diastolic blood pressures, a higher educational level, and a higher MMSE score at baseline. As expected, lipid fractions were correlated with one another, the strongest correlations being observed between LDL-C and TC, and between TG and HDL-C (S1 Table).

**Table 1:** Baseline characteristics of 3C participants.

Characteristic	Individuals included in analyses, *n =* 7,470	Individuals not included in analyses*, *n =* 1,824	*p*-Value
**Age, years**	73.83 ± 5.32	76.29 ± 6.30	<0.0001
**Women**	4,558 (61.02)	1,086 (59.54)	0.2466
Triglycerides, mmol/l**	1.25 ± 0.61	1.34 ± 0.68^‡^	<0.0001
**Total cholesterol concentration, mmol/l**	5.82 ± 0.97	5.81 ± 1.08^‡^	0.6979
**HDL-C concentration, mmol/l**	1.62 ± 0.40	1.55 ± 0.43^‡^	<0.0001
**LDL-C concentration, mmol/l**	3.63 ± 0.85	3.64 ± 0.93^‡^	0.7313
**Hypercholesterolemia**	4,258 (57.00)	845 (59.97)^‡^	0.0386
**Lipid-lowering drug intake**	2,298 (30.76)	495 (27.14)	0.0025
**Systolic blood pressure, mm Hg**	146.34 ± 21.64	147.70 ± 22.63	0.0207
**Diastolic blood pressure, mm Hg**	82.36 ± 11.20	81.58 ± 11.97	0.0128
**Hypertension**	5,732 (76.73)	1,473 (81.34)	<0.0001
**Ever smoker**	2,890 (38.70)	696 (38.20)	0.6921
**Diabetes**	696 (9.35)	216 (16.65)^‡^	<0.0001
**BMI**	25.65 ± 4.00	25.68 ± 4.32	0.7477
***APOE*ε4 carrier**	1,489 (20.03)	289 (23.27)^‡^	0.0089
**History of cardiovascular disease**	661 (8.85)	247 (13.59)	<0.0001
**Educational level**			<0.0001^§^
No education	107 (1.43)	91 (5.02)	
Primary school	2,194 (29.37)	710 (39.14)	
No education/primary school categories combined	2,301 (30.80)	801 (44.16)	
Secondary school with certificate of vocational aptitude	993 (13.29)	319 (17.58)	
Secondary school with secondary education certificate	1,270 (17.00)	210 (11.58)	
Secondary school categories combined	2,263 (30.29)	529 (29.16)	
Baccalaureate or equivalent	1,049 (14.04)	160 (8.82)	
University or equivalent	1,857 (24.86)	324 (17.86)	
**Mini-Mental State Examination score**^†^	27.64 ± 1.55	25.11 ± 3.40	<0.0001

Values are mean ± standard deviation or number (percentage). Body mass index (BMI) was calculated as the ratio of weight (in kilograms) to the square of height (in meters). Diabetes was defined as fasting blood glucose ≥ 7 mmol/l or antidiabetic drug intake or medical history of diabetes. Hypercholesterolemia was defined as fasting total cholesterol ≥ 6.2 mmol/l or lipid-lowering drug intake. Hypertension was defined as systolic blood pressure ≥ 140 mm Hg or diastolic blood pressure ≥ 90 mm Hg or antihypertensive drug intake.

*Excluded for brain tumor, prevalent dementia, or Mini-Mental State Examination score < 24 at baseline, or missing data for lipid concentrations, educational level, Mini-Mental State Examination score, or follow-up data.

**Triglycerides were log-transformed for analyses.

^‡^>10% missing data.

^§^For the comparison of individuals with either no education or primary school versus the other categories.

^†^Number of good answers.

HDL-C, high-density lipoprotein cholesterol; LDL-C, low-density lipoprotein cholesterol.

Over the follow-up period (mean [SD] = 7.9 [3.6] y), 779 participants developed dementia, of which 532 were classified as AD, 95 as mixed dementia and 59 as vascular dementia (due to small numbers, mixed and vascular dementia were considered together), and 93 as other types of dementia (excluded). Higher LDL-C and TC concentrations at baseline were significantly associated with an increased risk of AD, and these associations were maintained after accounting for the competing risk of death and after adjustment for vascular risk factors, even after replacing lipid-lowering drug intake by statin intake (Tables 2, S2 and S3). Effect size and significance level were reduced after additionally adjusting for *APOE*ε4 genotype, but remained significant for the association of LDL-C and AD and nominally significant for the association of TC and AD (*p =* 0.0171, not significant according to our statistical criterion allowing for multiple testing) (Tables 2 and S3). Higher TG concentration at baseline was associated with a significantly increased incidence of all and mixed or vascular dementia, even when accounting for the competing risk of death, except for the association with mixed or vascular dementia, which became nominally significant (*p =* 0.0188) (Tables 2 and S3). These associations became nonsignificant after adjusting for vascular risk factors, with or without additional adjustment for *APOE*ε4 genotype, and replacing lipid-lowering drug intake by statin intake did not modify these results (Tables 2, S2 and S3).

**Table 2:** Associations between baseline lipid concentrations and 13-y incident dementia using Cox models.

Model and outcome	TGs			HDL-C			LDL-C			TC		
	*n/N*	HR (95% CI)	*p*-Value	*n/N*	HR (95% CI)	*p*-Value	*n/N*	HR (95% CI)	*p*-Value	*n/N*	HR (95% CI)	*p*-Value
**Model 1: adjusted for sex, education, center, education × log(age)**^†^												
All dementia	778/7,466	1.11 (1.03, 1.19)	0.0044	779/7,467	0.92 (0.86, 1.00)	0.0431	776/7,440	1.07 (1.00, 1.15)	0.0511	779/7,470	1.07 (1.00, 1.15)	0.0563
Alzheimer disease	531/7,466	1.06 (0.97, 1.16)	0.1693	532/7,467	0.95 (0.87, 1.04)	0.2580	529/7,440	1.13 (1.04, 1.22)	0.0045	532/7,470	1.12 (1.03, 1.22)	0.0072
Mixed or vascular dementia	154/7,466	1.21 (1.04, 1.41)	0.0163	154/7,467	0.90 (0.76, 1.07)	0.2260	154/7,440	0.99 (0.85, 1.16)	0.9140	154/7,470	1.01 (0.86, 1.19)	0.8906
**Model 2: adjusted for sex, education, center, education × log(age)**^†^**, vascular risk factors**^‡^												
All dementia	761/7,376	1.05 (0.97, 1.15)	0.2424	761/7,376	0.98 (0.90, 1.08)	0.6924	761/7,376	1.10 (1.02, 1.18)	0.0153	762/7,402	1.09 (1.01, 1.18)	0.0263
Alzheimer disease	522/7,376	1.00 (0.90, 1.11)	0.9909	522/7,376	0.97 (0.87, 1.09)	0.6250	522/7,376	1.16 (1.06, 1.27)	0.0009	523/7,402	1.15 (1.05, 1.26)	0.0028
Mixed or vascular dementia	150/7,376	1.16 (0.95, 1.41)	0.1406	150/7,376	1.05 (0.85, 1.29)	0.6570	150/7,376	0.98 (0.83, 1.17)	0.8620	150/7,402	1.01 (0.85, 1.20)	0.9178
**Model 3: adjusted for sex, education, center, education × log(age)**^†^**, vascular risk factors**^‡^**, *APOEε4* carrier status**												
All dementia	755/7,344	1.07 (0.98, 1.17)	0.1374	755/7,344	0.99 (0.91, 1.09)	0.8905	755/7,344	1.06 (0.99, 1.15)	0.1123	756/7,369	1.06 (0.98, 1.15)	0.1258
Alzheimer disease	518/7,344	1.02 (0.91, 1.13)	0.7669	518/7,344	0.99 (0.88, 1.10)	0.8280	518/7,344	1.12 (1.03, 1.23)	0.0110	519/7,369	1.12 (1.02, 1.23)	0.0171
Mixed or vascular dementia	149/7,344	1.17 (0.96, 1.42)	0.1206	149/7,344	1.07 (0.87, 1.32)	0.5050	149/7,344	0.96 (0.81, 1.14)	0.6130	149/7,369	0.99 (0.83, 1.18)	0.9101

Results are given per standard deviation of lipid fraction (TG = 0.417; LDL = 0.854; HDL = 0.401; TC = 0.974).

^†^Age represents age at last follow-up or dementia.

^‡^Vascular risk factors for HDL-C, LDL-C, TGs: the three lipid fractions, body mass index, antihypertensive drug intake, systolic blood pressure, lipid-lowering drug intake, smoking status, diabetes, history of cardiovascular disease, antihypertensive drug intake × log(age). Vascular risk factors for TC: TGs, body mass index, antihypertensive drug intake, systolic blood pressure, lipid-lowering drug intake, smoking status, diabetes, history of cardiovascular disease, antihypertensive drug intake × log(age);

HDL-C, high-density lipoprotein cholesterol; HR, hazard ratio; LDL-C, low-density lipoprotein cholesterol; TC, total cholesterol; TGs, triglycerides.

We ran secondary association analyses stratified on dementia risk factors and putative effect modifiers. HRs were in the same direction when stratifying by participants’ *APOE*ε4 carrier status, use of lipid-lowering drugs, sex, or educational level (based on a dichotomized variable comparing individuals with no education or primary school versus the rest), and there was no significant interaction with any of these variables (S4–S10 Tables). Adjustment for use of lipid-lowering drugs also yielded similar findings in sex-specific analyses (S6 Table). Moreover, when using a three-class variable to account for the effect of lipid-lowering drug intake on lipid concentrations, higher LDL-C concentrations were significantly associated with an increased risk of AD both in participants not on lipid-lowering drugs and in participants on lipid-lowering drugs with high TC concentrations, but not in participants on lipid-lowering drugs with normal TC concentrations, though the interaction was only nominally significant (*p =* 0.0294) (S10 Table). However, there was a nominally significant interaction with age for the relationship of TC and all dementia (*p =* 0.0313) or AD (*p =* 0.0468), significant associations being observed only in the group aged at least 73.1 y at baseline (S4 Table).

**Fig 1:**
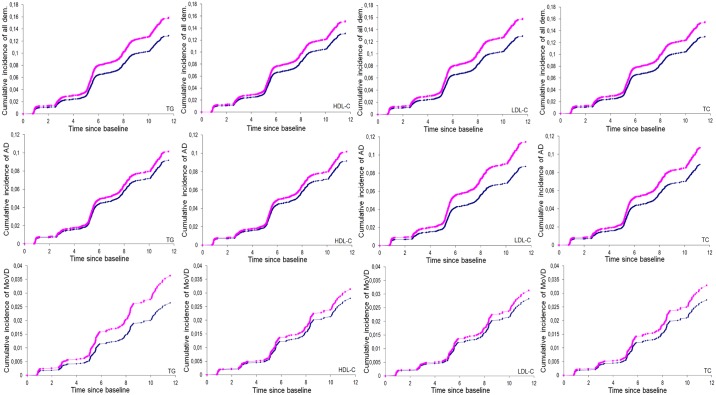
Cumulative incidence of dementia according to lipid concentrations (triglycerides, HDL-C, LDL-C, total cholesterol). These graphs are based on age-, sex-, and education-adjusted Cox models (from which indicated *p*-values were extracted). TG: blue, <1.44 mmol/l (women) or <1.55 mmol/l (men), versus pink, ≥1.44 mmol/l (women) or ≥1.55 mmol/l (men); *p*-values: *p =* 0.0046 (all dementia), *p =* 0.2745 (AD), *p =* 0.0623 (mixed or vascular dementia). HDL-C: blue, >1.46 mmol/l (women) or >1.20 mmol/l (men), versus pink, ≤1.46 mmol/l (women) or ≤1.20 (men); *p*-values: *p =* 0.0528 (all dementia), *p =* 0.2599 (AD), *p =* 0.5250 (mixed or vascular dementia). LDL-C: blue, <4.25 mmol/l (women) or <4.00 mmol/l (men), versus pink, ≥4.25 mmol/l (women) or ≥4.00 mmol/l (men); *p*-values: *p =* 0.0065 (all dementia), *p =* 0.0028 (AD), *p =* 0.5691 (mixed or vascular dementia).TC: blue, <6.60 mmol/l (women) or <6.10 mmol/l (men), versus pink, ≥6.60 mmol/l (women) or ≥6.10 mmol/l (men); *p*-values: *p =* 0.0210 (all dementia), *p =* 0.0383 (AD), *p =* 0.3259 (mixed or vascular dementia). AD, Alzheimer disease; dem., dementia; HDL-C, high-density lipoprotein cholesterol; LDL-C, low-density lipoprotein cholesterol; MoVD, mixed or vascular dementia; TC, total cholesterol; TG, triglycerides.

No deviation from linearity was found for all significant associations of LDL-C, TC, and TG with incident dementia in Cox models. When studying the associations using lipid concentrations in sex-specific quartiles, individually, the top quartile (or bottom quartile for HDL-C) was significantly associated with an increased risk of dementia (AD for LDL-C and TC, all dementia for TG, and all dementia for HDL-C; S11 Table) compared to the bottom reference quartile (top quartile for HDL-C). Age-, sex-, and education-adjusted cumulative dementia incidence graphs stratified on the top (or bottom for HDL-C) sex-specific quartile of each lipid fraction (versus the three other quartiles) are represented in Fig 1. All associations reported as significant for continuous lipid fractions also showed significant trend tests relating sex-specific lipid quartiles to dementia risk, except for the relationship between LDL-C and AD, for which the trend was only nominally significant (*p =* 0.0420).

## Discussion

In a cohort comprising 7,470 community-dwelling older persons, of whom 779 developed dementia over 13 y, associations of higher LDL-C, TC, and TGs with incident dementia were observed. When looking at dementia subtypes, we observed distinct patterns for the different lipid fractions: higher baseline LDL-C and TC concentrations were associated with AD, while higher baseline TG concentrations were associated with vascular or mixed dementia. For LDL-C and TC, associations were unchanged after accounting for vascular risk factors, but were attenuated after adjusting for *APOE*ε4 carrier status. For TGs, the results were no longer significant after adjusting for vascular risk factors. HDL-C concentrations were not associated with risk of incident dementia or its subtypes.

The current literature concerning associations between lipid concentrations and incident dementia risk is conflicting. Several studies reported a significant association of higher TC concentrations with increased risk of all dementia [6], AD [7–10], or dementia mortality [21,22]. However, two studies (*n =* 3,264 and 382) reported inverse associations with AD [18,19], and a number of publications reported no significant association with all dementia [4,13–15,45], AD [11,12,14,15,45,46], or vascular dementia risk [12,15,18]. Higher LDL-C concentrations were associated with an increased risk of vascular dementia in one study [11], whereas most studies reported no significant associations with all dementia [45], AD [11,12,20,45], or vascular dementia risk [12]. Most studies reported no significant relationship between TG concentration and risk of dementia [12,16,17,47], AD [5,12,16,47,48], or mixed or vascular dementia [5,12,16,17,47]. Although two studies have reported an association of lower HDL-C concentration with increased risk of all dementia (*n =* 337) [49] or AD (*n =* 1,130) [20], the vast majority reported no significant associations with all dementia [5,14,16,17,47], AD [5,11,12,14,16,17,47], or vascular dementia risk [5,12,16,17,47].

Considering that the mean age at dementia diagnosis is around 85 y [50], and that the pathological processes leading to dementia start many years before clinical diagnosis [51], the age when lipid concentrations are measured, the follow-up duration, and the age at the end of follow-up are critical when exploring the relation between lipid fractions and dementia risk. In the literature, studies reporting significant associations between lipid concentrations and dementia risk were mostly studies where lipid concentrations were measured in midlife and/or individuals were followed-up for a long period of time until advanced late life [6–10]. In our study, where lipid concentrations were assayed at the age of 65 y or older, we observed a nominally significant interaction with age at admission, with associations of lipid fractions with dementia risk being significant only in the older half of our sample (mean age 78.2 ± 3.9 y at baseline) [50].

The mechanisms underlying the association between lipid fractions and dementia risk are speculative. First, associations between lipid fractions and dementia risk may be directly mediated by cerebrovascular disease. We previously reported that higher TG concentrations were associated with a higher risk and burden of MRI markers for cerebral small vessel disease (WMHs and lacunar infarcts) [23]. Extensive WMHs and lacunar infarcts were repeatedly shown to be associated with dementia risk [26,52], and especially with mixed or vascular dementia risk [26]. This is consistent with the association pattern between TG concentrations and dementia we observed, including a significant association with mixed or vascular dementia that lost significance after adjusting for vascular risk factors (adjusting for MRI markers of cerebral small vessel disease was not possible as MRI measures were available only in a much smaller subset of the study population). Along the same lines, higher LDL-C concentrations were associated with increased risk of AD, but not mixed or vascular dementia, in line with the absence of significant association between higher LDL-C concentrations and MRI markers of cerebral small vessel disease [23].

Second, the observed associations may be an indirect marker of the relation between dementia and the *APOE*ε4 allele, as APOE is both a key player in lipid metabolism [53] and one of the strongest risk factors for dementia and AD (*APOE*ε4 allele*)* [53,54]. The attenuation of the association between LDL-C or TC and dementia risk after adjusting for *APOE*ε4 status (both in terms of effect size and significance) supports this hypothesis. Other genetic factors may also play a role, such as variants in *ATP binding cassette subfamily A member 1* (*ABCA1)* that are genome-wide significant risk variants for both LDL-C concentration and AD; additional AD risk variants are located near genes involved in lipid metabolism, such as *sortilin-related receptor L1* (*SORL1*), *clusterin* (*CLU*), *ATP binding cassette subfamily A member 7* (*ABCA7*), *phosphatidylinositol-binding clathrin assembly protein* (*PICALM*), and *bridging integrator 1* (*BIN1*) [55,56]. Whether variants in the aforementioned genes share associations with lipid fractions and AD via independent genetic effects on both phenotypes (pleiotropy) [56] or rather reflect true causal associations between lipid fractions and AD is a debated question. Appropriately designed intervention studies may be required to address this question.

To date, results of available experimental studies do not allow us to draw any conclusion on the existence of a causal relation between dyslipidemia and dementia risk. Two trials have examined the impact of lipid-lowering drugs, specifically statins, on dementia or cognitive decline [57,58]. The PROSPER trial (pravastatin versus placebo) showed no difference in global cognitive function (MMSE) at 4 y between patients on treatment and those on placebo [59]. Likewise, there was no difference in the incidence of dementia or in cognitive performance after 5 y in the HPS trial (simvastatin versus placebo) [60]. However, in both these trials, dementia or cognitive decline were only secondary outcomes, and follow-up was relatively short. In the present study, the cumulative incidence curves of dementia seem to start separating mostly after 5 y between participants with high versus low LDL-C and TC concentrations. This suggests that longer term reduction of cholesterol concentrations may be required to observe a difference, which may be challenging to implement in practice, although use of surrogate endpoints such as change in intermediate markers may be a way to circumvent this difficulty. Another challenge is that lipid-lowering drugs do not lower all lipid fractions homogeneously [61,62]. Of note, longitudinal epidemiological studies have also failed to show a significant association of lipid-lowering drugs with dementia risk in a recent systematic review [63], in line with our findings, although some studies have reported a beneficial effect [64,65], with the limitations inherent to studying drug effects in an observational setting and with limited duration of follow-up in most studies, although mostly longer than in the trials.

Strengths of our study include the large size of this population-based sample, with prospective diagnosis of dementia over up to 13 y, gathering one of the largest numbers of incident cases in a single study, with expert-panel-based validation and subtyping of dementia cases. Of note, this sample of non-institutionalized volunteers is not perfectly representative of the French general population of the same age range, as individuals taking part in a cohort study with regular follow-up examinations are more likely to be health-conscious and to have fewer risk factors than individuals who do not. This limitation is common to all prospective population-based studies, regardless of the sampling method used. However, as suggested by the PAQUID study, population-based samples may become more representative of the general population after a long follow-up period [66]. Another limitation is the fairly old age of our population at baseline, when lipid fractions were measured. Indeed, lipid concentrations can be modified by behavioral changes, the presence of comorbidities, or initiation of lipid-lowering therapy. Midlife lipid concentrations, which better reflect exposure to dyslipidemia over a lifespan, were not available [28,67]. We were also limited by the fact that exposures (lipid fractions and lipid-lowering drugs) vary during follow-up, and this was not accounted for in the present study, since follow-up data on exposures were available only on a very limited and selected subsample of the population from Dijon, at 4 y of follow-up (those who underwent both baseline and follow-up brain MRIs, *n =* 1,564, with 109 incident dementia cases). Moreover, our study may be limited by interval censoring because of the way age at dementia diagnosis was assessed (median of the interval from the last follow-up visit without dementia to the first follow-up visit with dementia) [68]. However, we did partially account for interval censoring by censoring non-demented participants at last date of follow-up instead of date of death (the latter yielded similar results in sensitivity analyses). Finally, we cannot exclude a possible survival bias, since individuals with major dyslipidemia may have died early of vascular disease, before being included in this study.

In summary, in a large population-based sample of older community-dwelling persons with 779 incident dementia cases occurring over up to 13 y of follow-up, we observed that higher LDL-C and TC concentrations were associated with an increased risk of AD. This association was independent of vascular risk factors and was attenuated but still significant after adjustment for *APOE*ε4 carrier status. This attenuation may indicate that these associations are, in part, an indirect marker of the relation between dementia and the *APOE*ε4 allele. If confirmed in independent samples, the observed independent associations may suggest that there is a rationale for better exploring the potential benefit of lipid-lowering drugs for preventing dementia in older community-dwelling persons by appropriately designed intervention studies.

## Supporting information

S1 Fig Selection flow chart for study sample. (TIF)

S1 Strobe Checklist STROBE checklist. (DOC)

S1 Table Correlations between lipid fractions. (DOCX)

S2 Table Associations between lipid concentrations at baseline and incident dementia over a 13-y period, using statin intake instead of lipid-lowering drug intake as adjustment. (DOCX)

S3 Table Associations between lipid concentrations at baseline and 13-y incident dementia using Cox models, accounting for competing risk of death. (DOCX)

S4 Table Association between lipid concentrations at baseline and incident dementia over a 13-y period, stratified by median age. (DOCX)

S5 Table Association between lipid concentrations at baseline and incident dementia over a 13-y period, stratified by sex. (DOCX)

S6 Table Association between lipid concentrations at baseline and incident dementia over a 13-y period, adjusted for lipid-lowering drug intake and stratified by sex. (DOCX)

S7 Table Association between lipid concentrations at baseline and incident dementia over a 13-y period, stratified by education. (DOCX)

S8 Table Association between lipid concentrations at baseline and incident dementia over a 13-y period, stratified by *APOE*ε4 carrier status. (DOCX)

S9 Table Association between lipid concentrations at baseline and incident dementia over a 13-y period, stratified by lipid-lowering drug intake. (DOCX)

S10 Table Associations between lipid concentrations at baseline and incident dementia over a 13-y period, stratified on a variable combining lipid-lowering drug intake and baseline total cholesterol concentration. (DOCX)

S11 Table Associations between lipid concentrations in sex-specific quartiles and 13-y incident dementia. (DOCX)

S1 Text Analysis plan. (DOCX)

## Abbreviations

3C Study: Three-City Study
AD: Alzheimer disease
BMI: body mass index
HDL-C: high-density lipoprotein cholesterol
HR: hazard ratio
LDL-C: low-density lipoprotein cholesterol
MMSE: Mini-Mental State Examination
SD: standard deviation
TC: total cholesterol
TG: triglyceride
WMH: white matter hyperintensity

## References

[1] Winblad B, Amouyel P, Andrieu S, Ballard C, Brayne C, Brodaty H, et al Defeating Alzheimer’s disease and other dementias: a priority for European science and society. Lancet Neurol. 2016;15(5):455–532. 10.1016/S1474-4422(16)00062-4 26987701

[2] Viswanathan A, Rocca WA, Tzourio C. Vascular risk factors and dementia: how to move forward? Neurology. 2009;72(4):368–74. 10.1212/01.wnl.0000341271.90478.8e 19171835 PMC2677504

[3] Norton S, Matthews FE, Barnes DE, Yaffe K, Brayne C. Potential for primary prevention of Alzheimer’s disease: an analysis of population-based data. Lancet Neurol. 2014;13(8):788–94. 10.1016/S1474-4422(14)70136-X 25030513

[4] de Bruijn RF, Bos MJ, Portegies ML, Hofman A, Franco OH, Koudstaal PJ, et al The potential for prevention of dementia across two decades: the prospective, population-based Rotterdam Study. BMC Med. 2015;13:132 10.1186/s12916-015-0377-5 26195085 PMC4509699

[5] Raffaitin C, Gin H, Empana JP, Helmer C, Berr C, Tzourio C, et al Metabolic syndrome and risk for incident Alzheimer’s disease or vascular dementia: the Three-City Study. Diabetes Care. 2009;32(1):169–74. 10.2337/dc08-0272 18945929 PMC2606808

[6] Whitmer RA, Sidney S, Selby J, Johnston SC, Yaffe K. Midlife cardiovascular risk factors and risk of dementia in late life. Neurology. 2005;64(2):277–81. 10.1212/01.WNL.0000149519.47454.F2 15668425

[7] Kivipelto M, Helkala EL, Laakso MP, Hanninen T, Hallikainen M, Alhainen K, et al Apolipoprotein E epsilon4 allele, elevated midlife total cholesterol level, and high midlife systolic blood pressure are independent risk factors for late-life Alzheimer disease. Ann Intern Med. 2002;137(3):149–55. 12160362 10.7326/0003-4819-137-3-200208060-00006

[8] Kivipelto M, Ngandu T, Fratiglioni L, Viitanen M, Kareholt I, Winblad B, et al Obesity and vascular risk factors at midlife and the risk of dementia and Alzheimer disease. Arch Neurol. 2005;62(10):1556–60. 10.1001/archneur.62.10.1556 16216938

[9] Notkola I-L, Sulkava R, Pekkanen J, Erkinjuntti T, Ehnholm C, Kivinen P, et al Serum total cholesterol, apolipoprotein E epsilon 4 allele, and Alzheimer’s disease. Neuroepidemiology. 1998;17(1):14–20. 9549720 10.1159/000026149

[10] Solomon A, Kivipelto M, Wolozin B, Zhou J, Whitmer RA. Midlife serum cholesterol and increased risk of Alzheimer’s and vascular dementia three decades later. Dement Geriatr Cogn Disord. 2009;28(1):75–80. 10.1159/000231980 19648749 PMC2814023

[11] Reitz C, Tang M-X, Luchsinger J, Mayeux R. Relation of plasma lipids to Alzheimer disease and vascular dementia. Arch Neurol. 2004;61(5):705–14. 10.1001/archneur.61.5.705 15148148 PMC2696387

[12] Yoshitake T, Kiyohara Y, Kato I, Ohmura T, Iwamoto H, Nakayama K, et al Incidence and risk factors of vascular dementia and Alzheimer’s disease in a defined elderly Japanese population: the Hisayama Study. Neurology. 1995;45(6):1161–8. 7783883 10.1212/wnl.45.6.1161

[13] Beydoun MA, Beason-Held LL, Kitner-Triolo MH, Beydoun HA, Ferrucci L, Resnick SM, et al Statins and serum cholesterol’s associations with incident dementia and mild cognitive impairment. J Epidemiol Community Health. 2011;65(11):949–57. 10.1136/jech.2009.100826 20841372 PMC3024452

[14] Li G, Shofer JB, Kukull WA, Peskind ER, Tsuang DW, Breitner JC, et al Serum cholesterol and risk of Alzheimer disease: a community-based cohort study. Neurology. 2005;65(7):1045–50. 10.1212/01.wnl.0000178989.87072.11 16217057

[15] Kalmijn S, Foley D, White L, Burchfiel CM, Curb JD, Petrovitch H, et al Metabolic cardiovascular syndrome and risk of dementia in Japanese-American elderly men. The Honolulu-Asia aging study. Arterioscler Thromb Vasc Biol. 2000;20(10):2255–60. 11031212 10.1161/01.atv.20.10.2255

[16] Muller M, Tang MX, Schupf N, Manly JJ, Mayeux R, Luchsinger JA. Metabolic syndrome and dementia risk in a multiethnic elderly cohort. Dement Geriatr Cogn Disord. 2007;24(3):185–92. 10.1159/000105927 17641531 PMC2268640

[17] Solfrizzi V, Scafato E, Capurso C, D’Introno A, Colacicco AM, Frisardi V, et al Metabolic syndrome and the risk of vascular dementia: the Italian Longitudinal Study on Ageing. J Neurol Neurosurg Psychiatry. 2010;81(4):433–40. 10.1136/jnnp.2009.181743 19965842

[18] Hayden KM, Zandi PP, Lyketsos CG, Khachaturian AS, Bastian LA, Charoonruk G, et al Vascular risk factors for incident Alzheimer disease and vascular dementia: the Cache County study. Alzheimer Dis Assoc Disord. 2006;20(2):93–100. 10.1097/01.wad.0000213814.43047.86 16772744

[19] Mielke MM, Zandi PP, Sjogren M, Gustafson D, Ostling S, Steen B, et al High total cholesterol levels in late life associated with a reduced risk of dementia. Neurology. 2005;64(10):1689–95. 10.1212/01.WNL.0000161870.78572.A5 15911792

[20] Reitz C, Tang MX, Schupf N, Manly JJ, Mayeux R, Luchsinger JA. Association of higher levels of high-density lipoprotein cholesterol in elderly individuals and lower risk of late-onset Alzheimer disease. Arch Neurol. 2010;67(12):1491–7. 10.1001/archneurol.2010.297 21149810 PMC3065942

[21] Strand BH, Langballe EM, Hjellvik V, Handal M, Næss Ø, Knudsen GP, et al Midlife vascular risk factors and their association with dementia deaths: results from a Norwegian prospective study followed up for 35 years. J Neurol Sci. 2013;324(1):124–30. 23146611 10.1016/j.jns.2012.10.018

[22] Alonso A, Jacobs DR, Menotti A, Nissinen A, Dontas A, Kafatos A, et al Cardiovascular risk factors and dementia mortality: 40 years of follow-up in the Seven Countries Study. J Neurol Sci. 2009;280(1):79–83. 19251275 10.1016/j.jns.2009.02.004

[23] Schilling S, Tzourio C, Dufouil C, Zhu Y, Berr C, Alperovitch A, et al Plasma lipids and cerebral small vessel disease. Neurology. 2014;83(20):1844–52. 10.1212/WNL.0000000000000980 25320101

[24] Carmelli D, Swan GE, Reed T, Wolf PA, Miller BL, DeCarli C. Midlife cardiovascular risk factors and brain morphology in identical older male twins. Neurology. 1999;52(6):1119–24. 10214731 10.1212/wnl.52.6.1119

[25] Park K, Yasuda N, Toyonaga S, Yamada SM, Nakabayashi H, Nakasato M, et al Significant association between leukoaraiosis and metabolic syndrome in healthy subjects. Neurology. 2007;69(10):974–8. 10.1212/01.wnl.0000266562.54684.bf 17538033

[26] Debette S, Beiser A, DeCarli C, Au R, Himali JJ, Kelly-Hayes M, et al Association of MRI markers of vascular brain injury with incident stroke, mild cognitive impairment, dementia, and mortality: the Framingham Offspring Study. Stroke. 2010;41(4):600–06. 10.1161/STROKEAHA.109.570044 20167919 PMC2847685

[27] 3C Study Group. Vascular factors and risk of dementia: design of the Three-City Study and baseline characteristics of the study population. Neuroepidemiology. 2003;22(6):316–25. 14598854 10.1159/000072920

[28] Stewart R, White LR, Xue QL, Launer LJ. Twenty-six-year change in total cholesterol levels and incident dementia: the Honolulu-Asia Aging Study. Arch Neurol. 2007;64(1):103–7. 10.1001/archneur.64.1.103 17210816

[29] Amieva H, Jacqmin-Gadda H, Orgogozo JM, Le Carret N, Helmer C, Letenneur L, et al The 9 year cognitive decline before dementia of the Alzheimer type: a prospective population-based study. Brain. 2005;128(Pt 5):1093–101. 10.1093/brain/awh451 15774508

[30] Amieva H, Le Goff M, Millet X, Orgogozo JM, Peres K, Barberger-Gateau P, et al Prodromal Alzheimer’s disease: successive emergence of the clinical symptoms. Ann Neurol. 2008;64(5):492–8. 10.1002/ana.21509 19067364

[31] Jacqmin-Gadda H, Fabrigoule C, Commenges D, Letenneur L, Dartigues JF. A cognitive screening battery for dementia in the elderly. J Clin Epidemiol. 2000;53(10):980–7. 11027929 10.1016/s0895-4356(00)00193-1

[32] American Psychiatric Association. Diagnostic and statistical manual of mental disorders. 4th ed Arlington (Virginia): American Psychiatric Association; 2000.

[33] Katz S, Ford AB, Moskowitz RW, Jackson BA, Jaffe MW. Studies of illness in the aged. The Index of ADL: a standardized measure of biological and psychosocial function. JAMA. 1963;185:914–9. 14044222 10.1001/jama.1963.03060120024016

[34] Lawton MP. Scales to measure competence in everyday activities. Psychopharmacol Bull. 1988;24(4):609–14. 3074322

[35] Rosow I, Breslau N. A Guttman health scale for the aged. J Gerontol. 1966;21(4):556–9. 5918309 10.1093/geronj/21.4.556

[36] McKhann G, Drachman D, Folstein M, Katzman R, Price D, Stadlan EM. Clinical diagnosis of Alzheimer’s disease: report of the NINCDS-ADRDA Work Group under the auspices of Department of Health and Human Services Task Force on Alzheimer’s Disease. Neurology. 1984;34(7):939–44. 6610841 10.1212/wnl.34.7.939

[37] Roman GC, Tatemichi TK, Erkinjuntti T, Cummings JL, Masdeu JC, Garcia JH, et al Vascular dementia: diagnostic criteria for research studies. Report of the NINDS-AIREN International Workshop. Neurology. 1993;43(2):250–60. 8094895 10.1212/wnl.43.2.250

[38] Friedewald WT, Levy RI, Fredrickson DS. Estimation of the concentration of low-density lipoprotein cholesterol in plasma, without use of the preparative ultracentrifuge. Clin Chem. 1972;18(6):499–502. 4337382

[39] Dufouil C, Richard F, Fievet N, Dartigues JF, Ritchie K, Tzourio C, et al APOE genotype, cholesterol level, lipid-lowering treatment, and dementia: the Three-City Study. Neurology. 2005;64(9):1531–8. 10.1212/01.WNL.0000160114.42643.31 15883313

[40] Thiebaut AC, Benichou J. Choice of time-scale in Cox’s model analysis of epidemiologic cohort data: a simulation study. Stat Med. 2004;23(24):3803–20. 10.1002/sim.2098 15580597

[41] Li J, Ji L. Adjusting multiple testing in multilocus analyses using the eigenvalues of a correlation matrix. Heredity (Edinb). 2005;95(3):221–7. 16077740 10.1038/sj.hdy.6800717

[42] Zissimopoulos JM, Barthold D, Brinton RD, Joyce G. Sex and race differences in the association between statin use and the incidence of Alzheimer disease. JAMA Neurol. 2017;74(2):225–32. 10.1001/jamaneurol.2016.3783 27942728 PMC5646357

[43] Leffondre K, Jager KJ, Boucquemont J, Stel VS, Heinze G. Representation of exposures in regression analysis and interpretation of regression coefficients: basic concepts and pitfalls. Nephrol Dial Transplant. 2014;29(10):1806–14. 10.1093/ndt/gft500 24366898

[44] Lin DY, Wei L-J, Ying Z. Checking the Cox model with cumulative sums of Martingale-based residuals. Biometrika. 1993;80(3):557–72.

[45] Ancelin ML, Ripoche E, Dupuy AM, Barberger-Gateau P, Auriacombe S, Rouaud O, et al Sex differences in the associations between lipid levels and incident dementia. J Alzheimers Dis. 2013;34(2):519–28. 10.3233/JAD-121228 23254630 PMC3966213

[46] Tan ZS, Seshadri S, Beiser A, Wilson PW, Kiel DP, Tocco M, et al Plasma total cholesterol level as a risk factor for Alzheimer disease: the Framingham Study. Arch Int Med. 2003;163(9):1053–7. 12742802 10.1001/archinte.163.9.1053

[47] Forti P, Pisacane N, Rietti E, Lucicesare A, Olivelli V, Mariani E, et al Metabolic syndrome and risk of dementia in older adults. J Am Geriatr Soc. 2010;58(3):487–92. 10.1111/j.1532-5415.2010.02731.x 20398117

[48] Warren MW, Hynan LS, Weiner MF. Lipids and adipokines as risk factors for Alzheimer’s disease. J Alzheimers Dis. 2012;29(1):151–7. 10.3233/JAD-2012-111385 22232009 PMC3732377

[49] Bonarek M, Barberger-Gateau P, Letenneur L, Deschamps V, Iron A, Dubroca B, et al Relationships between cholesterol, apolipoprotein E polymorphism and dementia: a cross-sectional analysis from the PAQUID study. Neuroepidemiology. 2000;19(3):141–8. doi: 26249 10705232 10.1159/000026249

[50] Satizabal CL, Beiser AS, Chouraki V, Chene G, Dufouil C, Seshadri S. Incidence of dementia over three decades in the Framingham Heart Study. N Engl J Med. 2016;374(6):523–32. 10.1056/NEJMoa1504327 26863354 PMC4943081

[51] Jack CR Jr, Knopman DS, Jagust WJ, Petersen RC, Weiner MW, Aisen PS, et al Tracking pathophysiological processes in Alzheimer’s disease: an updated hypothetical model of dynamic biomarkers. Lancet Neurol. 2013;12(2):207–16. 10.1016/S1474-4422(12)70291-0 23332364 PMC3622225

[52] Vermeer SE, Prins ND, den Heijer T, Hofman A, Koudstaal PJ, Breteler MM. Silent brain infarcts and the risk of dementia and cognitive decline. N Engl J Med. 2003;348(13):1215–22. 10.1056/NEJMoa022066 12660385

[53] Verghese PB, Castellano JM, Holtzman DM. Apolipoprotein E in Alzheimer’s disease and other neurological disorders. Lancet Neurol. 2011;10(3):241–52. 10.1016/S1474-4422(10)70325-2 21349439 PMC3132088

[54] Henderson AS, Easteal S, Jorm AF, Mackinnon AJ, Korten AE, Christensen H, et al Apolipoprotein E allele epsilon 4, dementia, and cognitive decline in a population sample. Lancet. 1995;346(8987):1387–90. 7475820 10.1016/s0140-6736(95)92405-1

[55] Lambert JC, Ibrahim-Verbaas CA, Harold D, Naj AC, Sims R, Bellenguez C, et al Meta-analysis of 74,046 individuals identifies 11 new susceptibility loci for Alzheimer’s disease. Nat Genet. 2013;45(12):1452–8. 10.1038/ng.2802 24162737 PMC3896259

[56] Ostergaard SD, Mukherjee S, Sharp SJ, Proitsi P, Lotta LA, Day F, et al Associations between potentially modifiable risk factors and Alzheimer disease: a Mendelian randomization study. PLoS Med. 2015;12(6):e1001841 10.1371/journal.pmed.1001841 26079503 PMC4469461

[57] McGuinness B, Craig D, Bullock R, Passmore P. Statins for the prevention of dementia. Cochrane Database Syst Rev. 2016;1:CD003160. 10.1002/14651858.CD003160.pub3 PMC9346344 26727124

[58] Debette S. Vascular risk factors and cognitive disorders. Rev Neurol (Paris). 2013;169(10):757–64. 24035574 10.1016/j.neurol.2013.07.022

[59] Trompet S, van Vliet P, de Craen AJ, Jolles J, Buckley BM, Murphy MB, et al Pravastatin and cognitive function in the elderly. Results of the PROSPER study. J Neurol. 2010;257(1):85–90. 10.1007/s00415-009-5271-7 19653027

[60] Heart Protection Study Collaborative Group. MRC/BHF Heart Protection Study of cholesterol lowering with simvastatin in 20,536 high-risk individuals: a randomised placebo-controlled trial. Lancet. 2002;360(9326):7–22. 10.1016/S0140-6736(02)09327-3 12114036

[61] Baigent C, Blackwell L, Emberson J, Holland LE, Reith C, Bhala N, et al Efficacy and safety of more intensive lowering of LDL cholesterol: a meta-analysis of data from 170,000 participants in 26 randomised trials. Lancet. 2010;376(9753):1670–81. 10.1016/S0140-6736(10)61350-5 21067804 PMC2988224

[62] Davidson MH, Stein EA, Dujovne CA, Hunninghake DB, Weiss SR, Knopp RH, et al The efficacy and six-week tolerability of simvastatin 80 and 160 mg/day. Am J Cardiol. 1997;79(1):38–42. 9024733 10.1016/s0002-9149(96)00742-4

[63] Power MC, Weuve J, Sharrett AR, Blacker D, Gottesman RF. Statins, cognition, and dementia-systematic review and methodological commentary. Nat Rev Neurol. 2015;11(4):220–9. 10.1038/nrneurol.2015.35 25799928 PMC4458855

[64] Haag MD, Hofman A, Koudstaal PJ, Stricker BH, Breteler MM. Statins are associated with a reduced risk of Alzheimer disease regardless of lipophilicity. The Rotterdam Study. J Neurol Neurosurg Psychiatry. 2009;80(1):13–7. 10.1136/jnnp.2008.150433 18931004

[65] Wolozin B, Wang SW, Li NC, Lee A, Lee TA, Kazis LE. Simvastatin is associated with a reduced incidence of dementia and Parkinson’s disease. BMC Med. 2007;5:20 10.1186/1741-7015-5-20 17640385 PMC1955446

[66] Helmer C, Peres K, Letenneur L, Guttierez-Robledo LM, Ramaroson H, Barberger-Gateau P, et al Dementia in subjects aged 75 years or over within the PAQUID cohort: prevalence and burden by severity. Dement Geriatr Cogn Disord. 2006;22(1):87–94. 10.1159/000093459 16710088

[67] Debette S, Seshadri S. Vascular risk factors and dementia revisited. J Neurol Neurosurg Psychiatry. 2009;80(11):1183–4. 10.1136/jnnp.2009.181289 19864653 PMC2837356

[68] Weuve J, Proust-Lima C, Power MC, Gross AL, Hofer SM, Thiebaut R, et al Guidelines for reporting methodological challenges and evaluating potential bias in dementia research. Alzheimers Dement. 2015;11(9):1098–109. 10.1016/j.jalz.2015.06.1885 26397878 PMC4655106

