# Supplementary figures and images for: Differential associations of plasma lipids with incident dementia and dementia subtypes in the 3C Study: A longitudinal, population-based prospective cohort study
Source: PLoS Med. 2017 Mar 28;14(3):e1002265. doi: 10.1371/journal.pmed.1002265 (PMC5369688; doi:10.1371/journal.pmed.1002265)

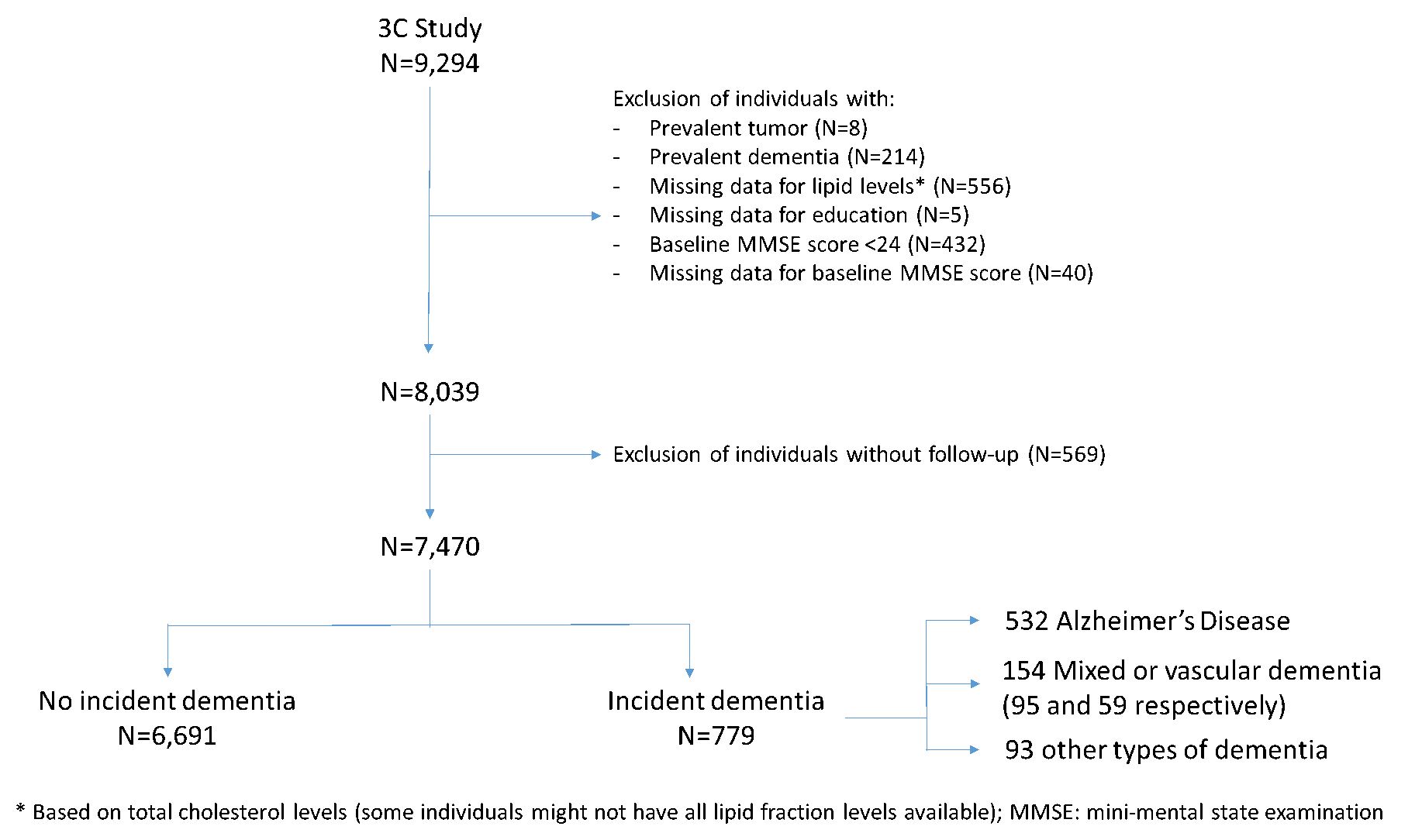

Supplement: S1 Fig — (TIF) [file pmed.1002265.s001.tif]
