# Supplementary material for: Differential associations of plasma lipids with incident dementia and dementia subtypes in the 3C Study: A longitudinal, population-based prospective cohort study
Source: PLoS Med. 2017 Mar 28;14(3):e1002265. doi: 10.1371/journal.pmed.1002265 (PMC5369688; doi:10.1371/journal.pmed.1002265)
Supplement: S1 Strobe Checklist — (DOC) [file pmed.1002265.s002.doc]

STROBE Statement—Checklist of items that should be included in reports of ***cohort studies***

|  | | Item No | Recommendation |
| --- | --- | --- | --- |
| **Title and abstract** | | 1 | (*a*) Indicate the study’s design with a commonly used term in the title or the abstract  **Main title**  **Differential associations of plasma lipids with incident dementia and subtypes in the 3C-Study: a longitudinal population-based prospective cohort study**  **Abstract, “Background” section**  **As lipid fractions represent easily modifiable targets, we examined the longitudinal relationship of baseline lipid fractions with 13-year incident dementia or its subtypes (Alzheimer’s disease [AD] and mixed or vascular dementia) in older community-persons.** |
|  |  |  | (*b*) Provide in the abstract an informative and balanced summary of what was done and what was found  **Abstract**  **Background: Vascular risk factors have been proposed as important targets for the prevention of dementia. As lipid fractions represent easily modifiable targets, we examined the longitudinal relationship of baseline lipid fractions with 13-year incident dementia or its subtypes (Alzheimer’s disease [AD] and mixed or vascular dementia) in older community-persons.**  **Methods and findings: 9,294 non-institutionalized persons aged 65+ years were recruited for the Three-City (3C) population-based cohort study from the electoral rolls of the cities of Dijon, Bordeaux, and Montpellier, France, between March 1999 and March 2001. Follow-up examinations were performed every 2 years after the baseline assessment. The study sample at baseline comprised 7,470 participants from the 3C study (mean age 73.8±5.3 years, 61.0% women), who were prospectively followed-up for up to 13 years. Fasting lipid fractions (triglycerides, high-density lipoprotein cholesterol [HDL-C], low-density lipoprotein cholesterol [LDL-C], total cholesterol) were studied as continuous variables and results were reported per standard deviation (SD) increase of each lipid fraction. Incident dementia and its subtypes were studied as censored variables using Cox models with age as time scale. Analyses were adjusted for sex, study center, and educational achievement, as well as vascular risk factors and apolipoprotein E (*APOE*) ε4 genotype in secondary analyses. We corrected for multiple testing, yielding a significance threshold of 0.0169. P-values above significance threshold but <0.05 were considered nominally significant.**  **During a mean (±SD) follow-up period of 7.9±3.6 years, 779 participants developed incident dementia (N=532 AD and N=154 mixed or vascular dementia). Higher LDL-C and total cholesterol concentrations at baseline were associated with an increased risk of AD (Hazard ratio[HR] per SD increase=1.13 [95% confidence interval 1.04-1.22], p=0.0045 and HR=1.12 [1.03-1.22], p=0.0072 respectively). These associations were substantially unchanged after adjustment for vascular risk factors, and attenuated after adjustment for APOE4 (HR per SD increase=1.12 [1.03-1.23], p=0.0110 and HR=1.12 [1.02-1.23], p=0.0171 respectively). Higher triglyceride concentrations at baseline were associated with increased risk of all dementia (HR per SD increase=1.11 [1.03-1.19], p=0.0044) and mixed or vascular dementia (HR=1.21 [1.04-1.41], p=0.0163). However, these associations disappeared after adjusting for vascular risk factors (HR=1.07 [0.98-1.17], p=0.1374, and HR=1.17 [0.96-1.42], p=0.1206 respectively). Main limitations include interval censoring of incident dementia cases, potential selective survival bias, and the fact that variation in lipid concentrations during follow-up could not be accounted for in the analyses.**  **Conclusions: In a large population-based sample of older community persons with up to 13 years of follow-up we observed that higher LDL-C and total cholesterol concentrations were associated with an increased risk of AD. This result was independent of vascular risk factors, and attenuated after adjustment for *APOE*ε4 carrier-status. Triglycerides and HDL-C concentrations were not associated with risk of incident dementia or its subtypes after accounting for vascular risk factors.** |
| Introduction | | | |
| Background/rationale | | 2 | Explain the scientific background and rationale for the investigation being reported  **Introduction (except last sentence)**  **Dementia refers to a group of neurological disorders characterized by memory loss, cognitive impairment and disability in activities of daily living. As the primary risk factor for dementia is old age, the prevalence of dementia is increasing dramatically, with aging populations worldwide. As no effective preventive treatment is currently available, the societal burden of dementia is huge and threatening to increase further. The most common form is Alzheimer’s disease (AD), a neurodegenerative disease representing 50-70% of dementia cases. Cerebrovascular disease is also a major contributor to dementia risk, often in conjunction with neurodegenerative lesions. Vascular risk factors have been proposed as important targets for the prevention of dementia, with around a third of AD cases being attributable to potentially modifiable risk factors, especially vascular risk factors, although trials have been inconclusive so far. As lipid fractions represent easily modifiable potential targets for prevention, exploring their relation with dementia risk is of major interest. So far published studies have shown inconsistent results, including associations of adverse lipid profile with increased dementia risk, absence of association or even inverse associations. Important differences between studies regarding the timing of the measurement of lipid fraction in relation to the diagnosis of dementia, the age at which plasma lipid concentrations are measured and the duration of follow-up might at least partly explain these discrepancies. Significant associations of high cholesterol concentrations with dementia, AD, or dementia death, are described mostly in studies where lipid concentrations are measured in midlife and/or participants are followed for a long period until advanced late life, hence with a long exposure to high cholesterol concentrations. In contrast, studies with lipid measurements in later life, or short follow-up periods not reaching ages at which dementia prevalence is highest, either do not observe any association, or sometimes inverse relations with dementia risk. Moreover, most studies have focused either on total cholesterol and LDL-C, or on TG and HDL-C, few studies have studied all fractions simultaneously in the same dataset. Interestingly, we and others have recently shown that lipid fractions, notably higher triglyceride concentrations, were significantly associated with white matter hyperintensity volume [WMHV] on brain MRI, a powerful predictor of dementia risk.** |
| Objectives | | 3 | State specific objectives, including any prespecified hypotheses  **Introduction, last sentence**  **In the present work we aimed to evaluate the relationship of lipid fractions (TG, HDL-C, LDL-C and TC) with incident dementia in a large cohort of community-dwelling older individuals with a long follow-up period over 13 years.** |
| Methods | | | |
| Study design | | 4 | Present key elements of study design early in the paper  **Methods and Materials, whole “study population” section**  **The 3C study is a longitudinal, population-based, prospective cohort study, described in detail elsewhere. Briefly, 9,294 non-institutionalized persons aged 65+ years were recruited from the electoral rolls of Dijon, Bordeaux, and Montpellier, France, between March 1999 and March 2001. Extensive follow-up examinations were performed every 2 years after the baseline assessment, comprising standardized questionnaires, clinical examinations, and detailed cognitive assessment. The third follow-up examination consisted of a self-questionnaire or a phone interview for participants who had refused to or could not fill in the questionnaire. We excluded participants with brain tumor (N=8), prevalent dementia (N=214), or with missing data for either lipid concentrations (N=556) or educational level (N=5). We also removed participants with Mini-Mental State Examination (MMSE) score less than 24 at baseline (N=432) (or with missing data for MMSE, N=40) as these individuals might have an undiagnosed dementia with lipid concentrations already impacted by metabolic changes secondary to the disease, thus leading to a sample of 8,039 participants at baseline. Incident dementia cases were prospectively ascertained over a 13-year follow-up period. Follow-up data on the outcome of interest was available in 7,470 of 8,039 participants (>92.9%), comprising our final study sample (see S1 Fig).** |
| Setting | | 5 | Describe the setting, locations, and relevant dates, including periods of recruitment, exposure, follow-up, and data collection  **Methods and Materials, first lines of the “study population” section**  **The 3C study is a longitudinal, population-based, prospective cohort study, described in detail elsewhere. Briefly, 9,294 non-institutionalized persons aged 65+ years were recruited from the electoral rolls of Dijon, Bordeaux, and Montpellier, France, between March 1999 and March 2001. Extensive follow-up examinations were performed every 2 years after the baseline assessment, comprising standardized questionnaires, clinical examinations, and detailed cognitive assessment. The third follow-up examination consisted of a self-questionnaire or a phone interview for participants who had refused to or could not fill in the questionnaire.**  **Page 9 – End of the “study population” section**  **Incident dementia cases were prospectively ascertained over a 13-year follow-up period.** |
| Participants | | 6 | (*a*) Give the eligibility criteria, and the sources and methods of selection of participants. Describe methods of follow-up  **Methods and Materials, whole “study population” section**  **The 3C study is a longitudinal, population-based, prospective cohort study, described in detail elsewhere. Briefly, 9,294 non-institutionalized persons aged 65+ years were recruited from the electoral rolls of Dijon, Bordeaux, and Montpellier, France, between March 1999 and March 2001. Extensive follow-up examinations were performed every 2 years after the baseline assessment, comprising standardized questionnaires, clinical examinations, and detailed cognitive assessment. The third follow-up examination consisted of a self-questionnaire or a phone interview for participants who had refused to or could not fill in the questionnaire. We excluded participants with brain tumor (N=8), prevalent dementia (N=214), or with missing data for either lipid concentrations (N=556) or educational level (N=5). We also removed participants with Mini-Mental State Examination (MMSE) score less than 24 at baseline (N=432) (or with missing data for MMSE, N=40) as these individuals might have an undiagnosed dementia with lipid concentrations already impacted by metabolic changes secondary to the disease, thus leading to a sample of 8,039 participants at baseline. Incident dementia cases were prospectively ascertained over a 13-year follow-up period. Follow-up data on the outcome of interest was available in 7,470 of 8,039 participants (>92.9%), comprising our final study sample (see S1 Fig).** |
|  |  |  | (*b*) For matched studies, give matching criteria and number of exposed and unexposed  **NA** |
| Variables | | 7 | Clearly define all outcomes, exposures, predictors, potential confounders, and effect modifiers. Give diagnostic criteria, if applicable  **Methods and Materials, “Outcome ascertainment and variables definition” section, “dementia ascertainment”, “laboratory testing” and “covariates” subsections**  ***Outcome ascertainment and variables definition***  **Dementia ascertainment**  **Dementia status was evaluated prospectively by an expert panel. In Bordeaux and Montpellier, all participants were examined by a neurologist. In Dijon, due to the large number of participants, a two-step procedure was used: (i) a careful neuropsychological evaluation carried out by trained psychologists; (ii) an examination by a neurologist for those who screened positively at step 1 based on MMSE and Isaacs’ Set Test, a measure of verbal fluency, and response rapidity, which consists of generating words belonging to given semantic categories (e.g. animal names) in 15 seconds. This test has been reported to show the earliest decline in the decade preceding dementia diagnosis. Cut-off scores were defined according to education level as previously described. Finally, in all centers, the examination and subtype classification of all suspected prevalent and incident dementia cases were performed by an independent committee of neurologists following the DSM-IV criteria. The final diagnosis and subtyping of dementia was made based on all available information, including data on cognitive functioning and daily activities, severity of cognitive disorders (Clinical Dementia Rating Scale), and where possible, hospitalization records, CT scans (which was most often used at the beginning of the follow-up period) and magnetic resonance images, and functional assessment which included assessment of disabilities using the Katz (activities of daily living), Lawton (instrumental activities of daily living) and Rosow and Breslau scales. Dementia subtypes include AD, vascular dementia and mixed dementia. Due to small numbers in the last two categories, these were pooled for analyses. Dementia subtyping was based, for AD, on the criteria of the National Institute of Neurological and Communication Disorders and Stroke–Alzheimer’s Disease and Related Disorders Association, and, for vascular dementia, on the criteria of the National Institute of Neurological Disorders and the Stroke-Association Internationale pour la Recherche et l’Enseignement en Neurosciences. Mixed dementia was defined as diagnosis of AD with either cerebrovascular lesions on brain imaging or a documented history of stroke and presence of prominent executive function in addition to an AD-type cognitive profile.**  **Laboratory testing**  **Centralized measurements of baseline fasting serum TC, HDL-C, and TG were performed using enzymatic methods. LDL-C was calculated with the Friedewald formula (LDL-cholesterol = total cholesterol – HDL-cholesterol - [triglycerides/2.2]) and was considered as missing for triglyceride values > 400 mg/dL (4.52 mmol/L).**  **Covariates**  **The following covariates were measured at baseline. Hypertension was defined by systolic blood pressure (SBP) ≥140mmHg, or diastolic blood pressure (DBP) ≥90mmHg, or use of antihypertensive drugs. Body mass index (BMI) was calculated as the ratio of weight (kg) to the square of height (m^2^). Diabetes was defined as fasting blood glucose ≥7mmol/L or antidiabetic drug intake or medical history of diabetes. Hypercholesterolemia was defined as fasting total cholesterol ≥6.2 mmol/L or use of any lipid-lowering drug (fibrates, statins or bile acid sequestrant). Smoking status was categorized as never, former and current smoker. History of cardiovascular disease was defined by a history of stroke, myocardial infarction, angina pectoris, or peripheral artery disease in both studies. Methods for genotyping the *APOE* epsilon polymorphism have been described previously. *APOE*ε4 carrier status was defined as the presence of at least one ε4 allele. Educational level at baseline was defined as a 6–class variable (no education; primary school; secondary school with certificate of vocational aptitude; secondary school with secondary education certificate; Baccalaureate or equivalent; University or equivalent). The MMSE provided a general assessment of global cognitive function.** |
| Data sources/ measurement | | 8* | Clearly define all outcomes, exposures, predictors, potential confounders, and effect modifiers. Give diagnostic criteria, if applicable  **Methods and Materials, “Outcome ascertainment and variables definition” section, “dementia ascertainment”, “laboratory testing” and “covariates” subsections**  ***Outcome ascertainment and variables definition***  **Dementia ascertainment**  **Dementia status was evaluated prospectively by an expert panel. In Bordeaux and Montpellier, all participants were examined by a neurologist. In Dijon, due to the large number of participants, a two-step procedure was used: (i) a careful neuropsychological evaluation carried out by trained psychologists; (ii) an examination by a neurologist for those who screened positively at step 1 based on MMSE and Isaacs’ Set Test, a measure of verbal fluency, and response rapidity, which consists of generating words belonging to given semantic categories (e.g. animal names) in 15 seconds. This test has been reported to show the earliest decline in the decade preceding dementia diagnosis. Cut-off scores were defined according to education level as previously described. Finally, in all centers, the examination and subtype classification of all suspected prevalent and incident dementia cases were performed by an independent committee of neurologists following the DSM-IV criteria. The final diagnosis and subtyping of dementia was made based on all available information, including data on cognitive functioning and daily activities, severity of cognitive disorders (Clinical Dementia Rating Scale), and where possible, hospitalization records, CT scans (which was most often used at the beginning of the follow-up period) and magnetic resonance images, and functional assessment which included assessment of disabilities using the Katz (activities of daily living), Lawton (instrumental activities of daily living) and Rosow and Breslau scales. Dementia subtypes include AD, vascular dementia and mixed dementia. Due to small numbers in the last two categories, these were pooled for analyses. Dementia subtyping was based, for AD, on the criteria of the National Institute of Neurological and Communication Disorders and Stroke–Alzheimer’s Disease and Related Disorders Association, and, for vascular dementia, on the criteria of the National Institute of Neurological Disorders and the Stroke-Association Internationale pour la Recherche et l’Enseignement en Neurosciences. Mixed dementia was defined as diagnosis of AD with either cerebrovascular lesions on brain imaging or a documented history of stroke and presence of prominent executive function in addition to an AD-type cognitive profile.**  **Laboratory testing**  **Centralized measurements of baseline fasting serum TC, HDL-C, and TG were performed using enzymatic methods. LDL-C was calculated with the Friedewald formula (LDL-cholesterol = total cholesterol – HDL-cholesterol - [triglycerides/2.2]) and was considered as missing for triglyceride values > 400 mg/dL (4.52 mmol/L).**  **Covariates**  **The following covariates were measured at baseline. Hypertension was defined by systolic blood pressure (SBP) ≥140mmHg, or diastolic blood pressure (DBP) ≥90mmHg, or use of antihypertensive drugs. Body mass index (BMI) was calculated as the ratio of weight (kg) to the square of height (m^2^). Diabetes was defined as fasting blood glucose ≥7mmol/L or antidiabetic drug intake or medical history of diabetes. Hypercholesterolemia was defined as fasting total cholesterol ≥6.2 mmol/L or use of any lipid-lowering drug (fibrates, statins or bile acid sequestrant). Smoking status was categorized as never, former and current smoker. History of cardiovascular disease was defined by a history of stroke, myocardial infarction, angina pectoris, or peripheral artery disease in both studies. Methods for genotyping the *APOE* epsilon polymorphism have been described previously. *APOE*ε4 carrier status was defined as the presence of at least one ε4 allele. Educational level at baseline was defined as a 6–class variable (no education; primary school; secondary school with certificate of vocational aptitude; secondary school with secondary education certificate; Baccalaureate or equivalent; University or equivalent). The MMSE provided a general assessment of global cognitive function.** |
| Bias | | 9 | Describe any efforts to address potential sources of bias  **Methods and materials, whole “statistical analyses” section**  ***Statistical analyses***  **To study the association between baseline lipid concentrations and 13-year incident dementia, we used Cox models, using age as time scale and birth as time origin. This allowed us to avoid the non-proportionality of dementia risk with age. Baseline age was the age at which subjects entered the cohort. Data were censored at the age of dementia diagnosis for cases (median of the interval of the last follow-up visit without dementia and the first follow-up visit with dementia) or at age at last follow-up for controls. This model also accounted for left-truncation and corrects for the bias introduced by including at baseline only individuals who did not develop dementia before inclusion. We verified the proportional hazard assumption using proportionality tests that assess the statistical significance of interaction terms between time (age at last follow-up or dementia occurrence) and the variables in the model. Since this assumption was not verified for educational achievement and antihypertensive drug intake, all Cox models also include an interaction term for these covariates with time (the interaction with antihypertensive drug intake being present only in models adjusted for vascular risk factors). Analyses were initially adjusted for study center, sex, educational level, and interaction between educational level and time (Model 1). Then additional adjustments were made for: i) vascular risk factors: BMI, systolic blood pressure, anti-hypertensive drug intake, interaction between anti-hypertensive drug intake and time, smoking status, diabetes, lipid-lowering drug intake and other lipid concentrations (analyses on total cholesterol were adjusted for triglycerides), cardiovascular disease (model 2) and ii) APOEε4 genotype (model 3). Lipid concentrations were studied as continuous variables and results were reported per increase in standard deviation (SD) of each lipid fraction. Of note, TG concentrations were log-transformed for analyses to remove skewness. When examining dementia subtypes, all subtypes that were not the primary outcome of interest were censored at the age of their diagnosis. We corrected for multiple testing by accounting for the number of independent lipid phenotypes examined, based on a previously described method, yielding a significance threshold of 0.0169 (based on a family-wise error rate of 0.05 and 3 independent tests) that was applied to all analyses. P-values >0.0169 but <0.05 were considered nominally significant.**  **In secondary analyses we first replaced overall lipid-lowering drug intake by statin intake in when adjusting for vascular risk factors. Second, we ran sensitivity analyses censoring non-demented individuals at the age at last follow-up or age at death, instead of age at last follow-up only, in order to account for competing risk of death. Third, we also performed Cox models using time-on-study as the time scale in order to generate cumulative incidence graphs, adjusting for age, sex and education, stratified on lipid concentrations (top quartile [or bottom for HDL-C], vs the rest). Fourth, we examined the robustness of our results by stratifying on APOEε4 carrier status, lipid-lowering drug intake, sex, median age, or educational level (using a dichotomized variable coding no education and primary school vs the rest) and formally tested for interaction with these variables. Fifth, we examined in greater depth the impact of lipid-lowering drug intake on associations. As the effect of lipid-lowering drugs on AD was reported to vary by sex, we also ran the analyses adjusted for lipid-lowering drug intake stratified by sex. Also, to take into account not only lipid-lowering drug intake but also the effect of lipid-lowering drugs on lipid concentrations, we stratified analyses on a 3-class variable crossing lipid-lowering drug intake and TC concentrations at baseline (i.e. no lipid-lowering intake; lipid-lowering intake and TC concentrations at baseline <6.2 mmol/L; lipid-lowering drug intake and TC concentrations at baseline ≥6.2 mmol/L). Sixth, we explored the relationship between sex-specific lipid quartiles and dementia risk, and linearity was assessed using the method based on Cox models as described above. The hazard ratio of each class of the lipid fraction divided in 4 classes, in each of which lipid concentrations were replaced by the value of the mean of the corresponding quartile minus that of the first quartile, was compared to the confidence interval of the hazard ratio of the corresponding quartile. The log-linearity hypothesis is acceptable if the hazard ratios calculated for the classes are included in the confidence intervals from the corresponding quartile. We also used plots of cumulative Martingale residuals against the continuous variable of interest (LDL-C, total cholesterol or TG).** |
| Study size | | 10 | Explain how the study size was arrived at  **Methods and Materials, second line of the “study population” section**  **Briefly, 9,294 non-institutionalized persons aged 65+ years were recruited from the electoral rolls of Dijon, Bordeaux, and Montpellier, France, between March 1999 and March 2001.**  **Page 8 and 9 (end of “study population” section)**  **We excluded participants with brain tumor (N=8), prevalent dementia (N=214), or with missing data for either lipid concentrations (N=556) or educational level (N=5). We also removed participants with Mini-Mental State Examination (MMSE) score less than 24 at baseline (N=432) (or with missing data for MMSE, N=40) as these individuals might have an undiagnosed dementia with lipid concentrations already impacted by metabolic changes secondary to the disease, thus leading to a sample of 8,039 participants at baseline. Incident dementia cases were prospectively ascertained over a 13-year follow-up period. Follow-up data on the outcome of interest was available in 7,470 of 8,039 participants (>92.9%), comprising our final study sample (see S1 Fig).** |
| Quantitative variables | | 11 | Explain how quantitative variables were handled in the analyses. If applicable, describe which groupings were chosen and why  **Abstract, “methods and findings” section**  **Fasting lipid fractions (triglycerides, high-density lipoprotein cholesterol [HDL-C], low-density lipoprotein cholesterol [LDL-C], total cholesterol) were studied as continuous variables and results were reported per standard deviation (SD) increase of each lipid fraction.**  **Page 12 “statistical analysis” section**  **Lipid levels were studied as continuous variables and results were reported per increase in standard deviation (SD) of each lipid fraction. Of note, TG levels were log-transformed for analyses to remove skewness.** |
| Statistical methods | | 12 | Describe any efforts to address potential sources of bias  **Methods and materials, whole “statistical analyses” section**  ***Statistical analyses***  **To study the association between baseline lipid concentrations and 13-year incident dementia, we used Cox models, using age as time scale and birth as time origin. This allowed us to avoid the non-proportionality of dementia risk with age. Baseline age was the age at which subjects entered the cohort. Data were censored at the age of dementia diagnosis for cases (median of the interval of the last follow-up visit without dementia and the first follow-up visit with dementia) or at age at last follow-up for controls. This model also accounted for left-truncation and corrects for the bias introduced by including at baseline only individuals who did not develop dementia before inclusion. We verified the proportional hazard assumption using proportionality tests that assess the statistical significance of interaction terms between time (age at last follow-up or dementia occurrence) and the variables in the model. Since this assumption was not verified for educational achievement and antihypertensive drug intake, all Cox models also include an interaction term for these covariates with time (the interaction with antihypertensive drug intake being present only in models adjusted for vascular risk factors). Analyses were initially adjusted for study center, sex, educational level, and interaction between educational level and time (Model 1). Then additional adjustments were made for: i) vascular risk factors: BMI, systolic blood pressure, anti-hypertensive drug intake, interaction between anti-hypertensive drug intake and time, smoking status, diabetes, lipid-lowering drug intake and other lipid concentrations (analyses on total cholesterol were adjusted for triglycerides), cardiovascular disease (model 2) and ii) APOEε4 genotype (model 3). Lipid concentrations were studied as continuous variables and results were reported per increase in standard deviation (SD) of each lipid fraction. Of note, TG concentrations were log-transformed for analyses to remove skewness. When examining dementia subtypes, all subtypes that were not the primary outcome of interest were censored at the age of their diagnosis. We corrected for multiple testing by accounting for the number of independent lipid phenotypes examined, based on a previously described method, yielding a significance threshold of 0.0169 (based on a family-wise error rate of 0.05 and 3 independent tests) that was applied to all analyses. P-values >0.0169 but <0.05 were considered nominally significant.**  **In secondary analyses we first replaced overall lipid-lowering drug intake by statin intake in when adjusting for vascular risk factors. Second, we ran sensitivity analyses censoring non-demented individuals at the age at last follow-up or age at death, instead of age at last follow-up only, in order to account for competing risk of death. Third, we also performed Cox models using time-on-study as the time scale in order to generate cumulative incidence graphs, adjusting for age, sex and education, stratified on lipid concentrations (top quartile [or bottom for HDL-C], vs the rest). Fourth, we examined the robustness of our results by stratifying on APOEε4 carrier status, lipid-lowering drug intake, sex, median age, or educational level (using a dichotomized variable coding no education and primary school vs the rest) and formally tested for interaction with these variables. Fifth, we examined in greater depth the impact of lipid-lowering drug intake on associations. As the effect of lipid-lowering drugs on AD was reported to vary by sex, we also ran the analyses adjusted for lipid-lowering drug intake stratified by sex. Also, to take into account not only lipid-lowering drug intake but also the effect of lipid-lowering drugs on lipid concentrations, we stratified analyses on a 3-class variable crossing lipid-lowering drug intake and TC concentrations at baseline (i.e. no lipid-lowering intake; lipid-lowering intake and TC concentrations at baseline <6.2 mmol/L; lipid-lowering drug intake and TC concentrations at baseline ≥6.2 mmol/L). Sixth, we explored the relationship between sex-specific lipid quartiles and dementia risk, and linearity was assessed using the method based on Cox models as described above. The hazard ratio of each class of the lipid fraction divided in 4 classes, in each of which lipid concentrations were replaced by the value of the mean of the corresponding quartile minus that of the first quartile, was compared to the confidence interval of the hazard ratio of the corresponding quartile. The log-linearity hypothesis is acceptable if the hazard ratios calculated for the classes are included in the confidence intervals from the corresponding quartile. We also used plots of cumulative Martingale residuals against the continuous variable of interest (LDL-C, total cholesterol or TG).** |
|  |  |  | (*b*) Describe any methods used to examine subgroups and interactions  **Methods and Materials, “statistical analysis” section, in paragraph 2**  **Fourth, we examined the robustness of our results by stratifying on *APOE*ε4 carrier status, lipid-lowering drug intake, sex, median age, or educational level (using a dichotomized variable coding no education and primary school vs the rest) and formally tested for interaction with these variables. Fifth, we examined in greater depth the impact of lipid-lowering drug intake on associations. As the effect of lipid-lowering drugs on AD was reported to vary by sex, we also ran the analyses adjusted for lipid-lowering drug intake stratified by sex. Also, to take into account not only lipid-lowering drug intake but also the effect of lipid-lowering drugs on lipid concentrations, we stratified analyses on a 3-class variable crossing lipid-lowering drug intake and TC concentrations at baseline (i.e. no lipid-lowering intake; lipid-lowering intake and TC concentrations at baseline <6.2 mmol/L; lipid-lowering drug intake and TC concentrations at baseline ≥6.2 mmol/L).** |
|  |  |  | (*c*) Explain how missing data were addressed  **NA** |
|  |  |  | (*d*) If applicable, explain how loss to follow-up was addressed  **NA** |
|  |  |  | (*e*) Describe any sensitivity analyses  **Methods and Materials, “statistical analyses” section, paragraph 2**  **In secondary analyses we first replaced overall lipid-lowering drug intake by statin intake in when adjusting for vascular risk factors. Second, we ran sensitivity analyses censoring non-demented individuals at the age at last follow-up or age at death, instead of age at last follow-up only, in order to account for competing risk of death. Third, we also performed Cox models using time-on-study as the time scale in order to generate cumulative incidence graphs, adjusting for age, sex and education, stratified on lipid concentrations (top quartile [or bottom for HDL-C], vs the rest). Fourth, we examined the robustness of our results by stratifying on *APOE*ε4 carrier status, lipid-lowering drug intake, sex, median age, or educational level (using a dichotomized variable coding no education and primary school vs the rest) and formally tested for interaction with these variables. Fifth, we examined in greater depth the impact of lipid-lowering drug intake on associations. As the effect of lipid-lowering drugs on AD was reported to vary by sex, we also ran the analyses adjusted for lipid-lowering drug intake stratified by sex. Also, to take into account not only lipid-lowering drug intake but also the effect of lipid-lowering drugs on lipid concentrations, we stratified analyses on a 3-class variable crossing lipid-lowering drug intake and TC concentrations at baseline (i.e. no lipid-lowering intake; lipid-lowering intake and TC concentrations at baseline <6.2 mmol/L; lipid-lowering drug intake and TC concentrations at baseline ≥6.2 mmol/L). Sixth, we explored the relationship between sex-specific lipid quartiles and dementia risk, and linearity was assessed using the method based on Cox models as described above. The hazard ratio of each class of the lipid fraction divided in 4 classes, in each of which lipid concentrations were replaced by the value of the mean of the corresponding quartile minus that of the first quartile, was compared to the confidence interval of the hazard ratio of the corresponding quartile. The log-linearity hypothesis is acceptable if the hazard ratios calculated for the classes are included in the confidence intervals from the corresponding quartile. We also used plots of cumulative Martingale residuals against the continuous variable of interest (LDL-C, total cholesterol or TG).** |
| Results | | | |
| Participants | | 13* | (a) Report numbers of individuals at each stage of study—eg numbers potentially eligible, examined for eligibility, confirmed eligible, included in the study, completing follow-up, and analysed  **Methods and Materials, “study population” section**  **The 3C study is a longitudinal, population-based, prospective cohort study, described in detail elsewhere. Briefly, 9,294 non-institutionalized persons aged 65+ years were recruited from the electoral rolls of Dijon, Bordeaux, and Montpellier, France, between March 1999 and March 2001. Extensive follow-up examinations were performed every 2 years after the baseline assessment, comprising standardized questionnaires, clinical examinations, and detailed cognitive assessment. The third follow-up examination consisted of a self-questionnaire or a phone interview for participants who had refused to or could not fill in the questionnaire. We excluded participants with brain tumor (N=8), prevalent dementia (N=214), or with missing data for either lipid concentrations (N=556) or educational level (N=5). We also removed participants with Mini-Mental State Examination (MMSE) score less than 24 at baseline (N=432) (or with missing data for MMSE, N=40) as these individuals might have an undiagnosed dementia with lipid concentrations already impacted by metabolic changes secondary to the disease, thus leading to a sample of 8,039 participants at baseline. Incident dementia cases were prospectively ascertained over a 13-year follow-up period. Follow-up data on the outcome of interest was available in 7,470 of 8,039 participants (>92.9%), comprising our final study sample (see S1 Fig).** |
|  |  |  | (b) Give reasons for non-participation at each stage  **Methods and Materials, “study population” section**  **The 3C study is a longitudinal, population-based, prospective cohort study, described in detail elsewhere. Briefly, 9,294 non-institutionalized persons aged 65+ years were recruited from the electoral rolls of Dijon, Bordeaux, and Montpellier, France, between March 1999 and March 2001. Extensive follow-up examinations were performed every 2 years after the baseline assessment, comprising standardized questionnaires, clinical examinations, and detailed cognitive assessment. The third follow-up examination consisted of a self-questionnaire or a phone interview for participants who had refused to or could not fill in the questionnaire. We excluded participants with brain tumor (N=8), prevalent dementia (N=214), or with missing data for either lipid concentrations (N=556) or educational level (N=5). We also removed participants with Mini-Mental State Examination (MMSE) score less than 24 at baseline (N=432) (or with missing data for MMSE, N=40) as these individuals might have an undiagnosed dementia with lipid concentrations already impacted by metabolic changes secondary to the disease, thus leading to a sample of 8,039 participants at baseline. Incident dementia cases were prospectively ascertained over a 13-year follow-up period. Follow-up data on the outcome of interest was available in 7,470 of 8,039 participants (>92.9%), comprising our final study sample (see S1 Fig).** |
|  |  |  | (c) Consider use of a flow diagram  **Due to technical reasons, it was not possible to insert the flow diagram here. We included a flow chart in the supporting information file (S1 Fig).** |
| Descriptive data | 14* | (a) Give characteristics of study participants (eg demographic, clinical, social) and information on exposures and potential confounders  **Results, Table 1 and legend**  **Table 1. Baseline characteristics of 3C participants**   |  | Individuals with MMSE≥24 at baseline | Individuals not included in analyses |  | | --- | --- | --- | --- | |  | N= 7,470 | N= 1,824 | p | | Age, years, mean ± SD | 73.83 ± 5.32 | 76.29 ± 6.30 | <0.0001 | | Women, N (%) | 4558 (61.02) | 1086 (59.54) | 0.2466 | | Triglycerides, mmol/L, mean ± SD** | 1.25 ± 0.61 | 1.34 ± 0.68 ‡ | <0.0001 | | Total cholesterol level, mmol/L, mean ± SD | 5.82 ± 0.97 | 5.81 ± 1.08 ‡ | 0.6979 | | HDL-cholesterol level, mmol/L, mean ± SD | 1.62 ± 0.40 | 1.55 ± 0.43 ‡ | <0.0001 | | LDL-cholesterol level, mmol/L, mean ± SD | 3.63 ± 0.85 | 3.64 ± 0.93 ‡ | 0.7313 | | Hypercholesterolemia, N (%) | 4258 (57.00) | 845 (59.97)‡ | 0.0386 | | Lipid-lowering drug intake, N (%) | 2298 (30.76) | 495 (27.14) | 0.0025 | | Systolic blood pressure, mmHg, mean ± SD | 146.34 ± 21.64 | 147.70 ± 22.63 | 0.0207 | | Diastolic blood pressure, mmHg, mean ± SD | 82.36 ± 11.20 | 81.58 ± 11.97 | 0.0128 | | Hypertension, N (%) | 5732 (76.73) | 1473 (81.34) | <0.0001 | | Ever smoker, N (%) | 2890 (38.70) | 696 (38.20) | 0.6921 | | Diabetes, N (%) | 696 (9.35) | 216 (16.65) ‡ | <0.0001 | | BMI, mean ± SD | 25.65 ± 4.00 | 25.68 ± 4.32 | 0.7477 | | *APOEε4* carriers, N (%) | 1489 (20.03) | 289 (23.27) ‡ | 0.0089 | | History of cardiovascular disease, N (%) | 661 (8.85) | 247 (13.59) | <0.0001 | | Educational level, years, N (%) |  |  | <0.0001§ | | 5 | 2301 (30.80) | 801 (44.16) |  | | no education / primary school | 107 (1.43)/ 2194 (29.37) | 91 (5.02)/ 710 (39.14) |  | | 6-9 | 2263 (30.29) | 529 (29.16) |  | | Secondary school with: certificate of vocational aptitude/ junior secondary education certificate | 993 (13.29)/ 1270 (17.00) | 319 (17.58)/ 210 (11.58) |  | | 12 (Baccalaureate) | 1049 (14.04) | 160 (8.82) |  | | >12 (University or equivalent) | 1857 (24.86) | 324 (17.86) |  | | Mini Mental State Examination†, mean ± SD | 27.64 ± 1.55 | 25.11 ± 3.40 | <0.0001 |   **Values are percentages (number) unless stated otherwise. † number of good answers; ‡ >10% missing data; § for the comparison of individuals with either no education or primary school versus the other categories; **Triglycerides were log-transformed for analyses; BMI: Body mass index, calculated as the ratio of weight (kg) to the square of height (m^2^); Diabetes: fasting blood glucose ≥7mmol/L or antidiabetic drug intake or medical history of diabetes; HDL: high-density lipoprotein; Hypercholesterolemia: fasting total cholesterol ≥6.2 mmol/L or use of any lipid-lowering drugs; Hypertension: systolic blood pressure (SBP) ≥140mmHg, or diastolic blood pressure (DBP) ≥90mmHg, or use of antihypertensive drugs; LDL: low-density lipoprotein; SD: standard deviation;** | |
|  |  | (b) Indicate number of participants with missing data for each variable of interest  **Results, caption of table 1**  **Values are percentages (number) unless stated otherwise. † number of good answers; ‡ >10% missing data; § for the comparison of individuals with either no education or primary school versus the other categories; **Triglycerides were log-transformed for analyses; BMI: Body mass index, calculated as the ratio of weight (kg) to the square of height (m^2^); Diabetes: fasting blood glucose ≥7mmol/L or antidiabetic drug intake or medical history of diabetes; HDL: high-density lipoprotein; Hypercholesterolemia: fasting total cholesterol ≥6.2 mmol/L or use of any lipid-lowering drugs; Hypertension: systolic blood pressure (SBP) ≥140mmHg, or diastolic blood pressure (DBP) ≥90mmHg, or use of antihypertensive drugs; LDL: low-density lipoprotein; SD: standard deviation;** | |
|  |  | (c) Summarise follow-up time (eg, average and total amount)  **Results, paragraph 2**  **Over the follow-up period (mean (SD) =7.9 (3.6) years), 779 participants developed dementia, of which 532 were classified as Alzheimer’s dementia, 95 as mixed dementia, 59 as vascular dementia (due to small numbers mixed and vascular dementia were studied together), and 93 as other types of dementia.** | |
| Outcome data | 15* | Report numbers of outcome events or summary measures over time  **Results, paragraph 2**  **Over the follow-up period (mean (SD) =7.9 (3.6) years), 779 participants developed dementia, of which 532 were classified as Alzheimer’s dementia, 95 as mixed dementia, 59 as vascular dementia (due to small numbers mixed and vascular dementia were studied together), and 93 as other types of dementia.** | |
| Main results | 16 | (*a*) Give unadjusted estimates and, if applicable, confounder-adjusted estimates and their precision (eg, 95% confidence interval). Make clear which confounders were adjusted for and why they were included  **Results, Table 2 and legend**  **Due to technical reasons, it was not possible to insert table 2 here.** | |
|  |  | (*b*) Report category boundaries when continuous variables were categorized  **NA for main analyses** | |
|  |  | (*c*) If relevant, consider translating estimates of relative risk into absolute risk for a meaningful time period  **NA** | |
| Other analyses | 17 | Report other analyses done—eg analyses of subgroups and interactions, and sensitivity analyses  **Results – in paragraph 2**  **Higher LDL-C and total cholesterol concentrations at baseline were significantly associated with an increased risk of AD, and these associations were maintained after accounting for competing risks of death or after adjustment for vascular risk factors, even after replacing lipid-lowering drug intake by statin intake (Table 2 and S2-S3 Tables). Effect size and significance level were reduced after additionally adjusting for *APOE*ε4 genotype, but remained significant for LDL-C and AD and nominally significant for total cholesterol and AD (p=0.0171, not significant according to our statistical criterion allowing for multiple testing) (Table 2, S3 Table). Higher triglyceride concentrations at baseline were associated with a significantly increased incidence of all and mixed or vascular dementia, even when accounting for competing risks of death, except for the association with mixed or vascular dementia which became nominally significant (p=0.0188) (Table 2, S3 Table). These associations became non-significant after adjusting for vascular risk factors, with or without additional adjustment for *APOE*ε4 genotype, and replacing lipid-lowering drug intake by statin intake did not modify these results (Table 2 and S2-S3 Tables).**  **Results, paragraph 3**  **We ran secondary association analyses stratified on dementia risk factors and putative effect modifiers. Hazard ratios were in the same direction according to participants’ *APOE*ε4 carrier status, use of lipid-lowering drugs, sex or educational level (based on a dichotomized variable comparing individuals with no education or primary school vs the rest) and there was no significant interaction with any of these variables (S4 to S10 Tables). Adjustment for lipid-lowering drugs also yielded similar findings in sex-specific analyses (S6 Table). Moreover, when using a three class variable to account for the effect of lipid-lowering drug intake on lipid concentrations, higher LDL-C concentrations were significantly associated with an increased risk of AD both in participants not on lipid-lowering drugs and in participants on lipid-lowering drugs with high TC concentrations, but no in participants on lipid-lowering drugs with normal TC concentrations, but the interaction was only nominally significant (p=0.0294) (S10 Table). However, there was a nominally significant interaction with median age on the relationship of total cholesterol and all dementia (p=0.0313) or AD (p=0.0468), significant associations being observed only in the group aged at least 73.1 at baseline (S4 Table).**  **No deviation from linearity was found for all significant associations of LDL-C, total cholesterol, and TG with incident dementia in Cox models. When studying the associations using lipid concentrations in sex-specific quartiles, individually, the top quartile (or bottom quartile for HDL-C), was significantly associated with increased risk of dementia (AD for LDL-C and total cholesterol, all dementia for TG, and all dementia for HDL-C, S11 Table) compared to the bottom reference quartile (top for HDL-C). Age, sex and education adjusted cumulative dementia incidence graphs stratified on the top (or bottom for HDL-C) sex-specific quartile of each lipid fraction (vs. the three other quartiles) are represented in Figure 1. All associations reported as significant for continuous lipid fractions also showed significant trend tests relating sex-specific lipid quartiles to dementia risk, except for the relationship between LDL-C and AD, for which the trend is only nominally significant (p=0.0420).** | |
| Discussion | | | |
| Key results | 18 | Summarise key results with reference to study objectives  **Discussion, paragraph 1**  **In a cohort comprising 7,470 community-dwelling older persons, of which 779 developed dementia over 13 years, associations of higher LDL-C, total cholesterol, and triglycerides with incident dementia were observed. When looking at dementia subtypes we observed distinct patterns for the different lipid fractions: higher baseline LDL-C and total cholesterol concentrations were associated with AD, while higher baseline TG concentrations were associated with vascular or mixed dementia. For LDL-C and total cholesterol, associations were unchanged after accounting for vascular risk factors, but attenuated after adjusting for *APOE*ε4 carrier-status. For TG, the results were no longer significant after adjusting for vascular risk factors. HDL-C concentrations were not associated with risk of incident dementia or its subtypes.** | |
| Limitations | 19 | Discuss limitations of the study, taking into account sources of potential bias or imprecision. Discuss both direction and magnitude of any potential bias  **Discussion, in paragraph 7**  **Of note, this sample of non-institutionalized volunteers is not perfectly representative of the French general population of the same age range, as individuals taking part in a cohort study with regular follow-up examinations are more likely to be health-conscious and have fewer risk factors than individuals who do not. This limitation is common to all prospective population-based studies, regardless of the sampling method used. However, as suggested by the Paquid study, population-based samples may become more representative of the general population after a long follow-up period. Another limitation is the fairly old age of our population at baseline when lipid fractions are measured. Indeed, lipid concentrations can be modified by behavioral changes, or by the presence of comorbidities, or initiation of lipid-lowering therapy. Midlife lipid concentrations, which better reflect exposure to dyslipidemia over a lifespan, were not available. We were also limited by the fact that exposures (lipid fractions, and lipid-lowering drugs) vary during follow-up and this was not accounted for in the present study, since this data was available only on a very limited and selected subsample of the population from Dijon, who underwent a brain MRI at baseline, and a second brain MRI at 4 years of follow-up (N=1,564 with 109 incident dementia cases). Moreover we may be limited by interval censoring due to the way age at dementia diagnosis was assessed (median of the interval of the last follow-up visit without dementia and the first follow-up visit with dementia). However, we did partially account for interval censoring by censoring non-demented participants at last date of follow-up instead of date of death (the latter yielded similar results in sensitivity analyses). Finally, we cannot exclude a possible survival bias, since individuals with major dyslipidemia may have died early of vascular disease, before being included in this study.** | |
| Interpretation | 20 | Give a cautious overall interpretation of results considering objectives, limitations, multiplicity of analyses, results from similar studies, and other relevant evidence  **Discussion, paragraphs 2 to 6**  **The current literature concerning associations between lipid concentrations and incident dementia risk is conflicting. Several studies reported a significant association of higher total cholesterol concentrations with increased risk of all dementia, AD, or dementia mortality. However, two studies (N=3,264 and 382) reported inverse associations with AD; and a number of publications did not report any significant association with all dementia, AD, or vascular dementia risk. Higher LDL-C concentrations were associated with an increased risk of vascular dementia in one study, whereas most studies reported no significant associations with all dementia, AD, or vascular dementia risk. Most studies did not report any significant relationship between TG concentrations and risk of dementia, AD, or mixed or vascular dementia. Although two studies have reported an association of lower HDL-C concentrations with increased risk of all dementia (N=337) or AD (N=1,130); the vast majority did not report any significant associations with all dementia, AD, or vascular dementia risk.**  **Considering that the mean age at dementia diagnosis is around 85 years, and that the pathological processes leading to dementia started many years before clinical diagnosis, the age when lipid concentrations are measured, the follow-up duration, and the age at the end of follow-up, are critical when exploring the relation between lipid fractions and dementia risk. In the literature, studies reporting significant associations between lipid concentrations and dementia risk were mostly carried out in individuals with midlife lipid concentration measurements and/or followed-up for a long period of time until advanced late-life. In our study, where lipid concentrations were assayed at the age of 65 years or older, we observed a nominally significant interaction with age at admission, associations of lipid fractions with dementia risk being significant only in the older half of our sample (mean age 78.2±3.9 years at baseline).**  **The mechanisms underlying the association between lipid fractions and dementia risk are speculative. First, associations between lipid fractions and dementia risk may be directly mediated by cerebrovascular disease. We previously reported that higher TG concentrations were associated with a higher risk and burden of MRI-markers for cerebral small vessel disease (white matter hyperintensities [WMH] and lacunar infarcts). Extensive WMH and lacunar infarcts were repeatedly shown to be associated with dementia risk, and especially with mixed or vascular dementia. This is consistent with the association pattern between TG concentrations and dementia we observed, including a significant association with mixed or vascular dementia that lost significance after adjusting for vascular risk factors (adjusting for MRI-markers of cerebral small vessel disease was not possible as MRI measures were available only in a much smaller subset of the study population). Along the same lines, higher LDL-C concentrations were associated with increased risk of AD, but not mixed or vascular dementia, in line with the absence of significant association between higher LDL-C concentrations and MRI-markers of cerebral small vessel disease.**  **Second, the observed associations may be an indirect marker of the relation between dementia and the *APOEε4* allele, as APOE is both a key player in lipid metabolism and one of the strongest risk factors for dementia and AD (*APOEε4* allele*)*. The attenuation of the association between LDL-C or total cholesterol and dementia risk after adjusting for *APOEε4* status (both in terms of effect size and significance) supports this hypothesis. Other genetic factors may also play a role, such as variants in ATP binding cassette subfamily A member 1 (*ABCA1)* that are genome-wide significant risk variants for both LDL-C concentrations and AD; additional AD risk variants are located near genes involved in lipid metabolism, such as Sortilin-related receptor L1 (*SORL1*), clusterin (*CLU*), ATP binding cassette subfamily A member 7 (*ABCA7*), phosphatidylinositol-binding clathrin assembly protein (*PICALM*) and bridging integrator 1 (*BIN1*). Whether variants in the aforementioned genes share associations with lipid fractions and AD via independent genetic effects on both phenotypes (pleiotropy) or rather reflect true causal associations between lipid fractions and AD is a debated question. Appropriately designed interventional studies may be required to address this question.**  **To date, results of available experimental studies do not allow drawing any conclusion on the existence of a causal relation between dyslipidemia and dementia risk. Two trials have examined the impact of lipid-lowering drugs, specifically statins, on dementia or cognitive decline The PROSPER trial (pravastatin vs. placebo) showed no difference in global cognitive function (Mini-Mental State Examination) at 4 years between patients on treatment and those on placebo. Likewise, there was no difference in incidence of dementia or in cognitive performance after 5 years in the HPS trial (simvastatin vs. placebo). However, in both these trials, dementia or cognitive decline were only secondary outcomes and follow-up was relatively short. In the present study the cumulative incidence curves of dementia seem to start separating mostly after 5 years between participants with high vs. low LDL-C and total cholesterol concentrations. This suggests that longer term reduction of cholesterol concentrations may be required to observe a difference, which may be challenging to implement in practice, although use of surrogate endpoints such as change in intermediate markers may be a way to circumvent this difficulty. Another challenge is that lipid-lowering drugs do not lower all lipid fractions homogeneously. Of note, longitudinal epidemiological studies have also failed to show a significant association of lipid-lowering drugs with dementia risk in a recent systematic review, in line with our findings, although some studies have reported a beneficial effect, with the limitations inherent to studying drug effects in an observational setting, and limited duration of follow-up in most studies, although mostly longer than in the trials.** | |
| Generalisability | 21 | Discuss the generalisability (external validity) of the study results  **Discussion, in paragraph 7**  **Of note, this sample of non-institutionalized volunteers is not perfectly representative of the French general population of the same age range, as individuals taking part in a cohort study with regular follow-up examinations are more likely to be health-conscious and have fewer risk factors than individuals who do not. This limitation is common to all prospective population-based studies, regardless of the sampling method used. However, as suggested by the Paquid study, population-based samples may become more representative of the general population after a long follow-up period. Another limitation is the fairly old age of our population at baseline when lipid fractions are measured. Indeed, lipid concentrations can be modified by behavioral changes, or by the presence of comorbidities, or initiation of lipid-lowering therapy. Midlife lipid concentrations, which better reflect exposure to dyslipidemia over a lifespan, were not available. We were also limited by the fact that exposures (lipid fractions, and lipid-lowering drugs) vary during follow-up and this was not accounted for in the present study, since this data was available only on a very limited and selected subsample of the population from Dijon, who underwent a brain MRI at baseline, and a second brain MRI at 4 years of follow-up (N=1,564 with 109 incident dementia cases). Moreover we may be limited by interval censoring due to the way age at dementia diagnosis was assessed (median of the interval of the last follow-up visit without dementia and the first follow-up visit with dementia). However, we did partially account for interval censoring by censoring non-demented participants at last date of follow-up instead of date of death (the latter yielded similar results in sensitivity analyses). Finally, we cannot exclude a possible survival bias, since individuals with major dyslipidemia may have died early of vascular disease, before being included in this study.** | |
| Other information | | | |
| Funding | 22 | Give the source of funding and the role of the funders for the present study and, if applicable, for the original study on which the present article is based  **The 3-City Study is conducted under a partnership agreement among the Institut National de la Santé et de la Recherche Médicale (INSERM), the Victor Segalen–Bordeaux II University, and Sanofi-Aventis. The Fondation pour la Recherche Médicale funded the preparation and initiation of the study. The 3C Study is also supported by the Caisse Nationale Maladie des Travailleurs Salariés, Direction Générale de la Santé, Mutuelle Générale de l’Education Nationale (MGEN), Institut de la Longévité, Conseils Régionaux of Aquitaine and Bourgogne, Fondation de France, Caisse Nationale de Solidarité et Longévité (CNSA) and Ministry of Research–INSERM Programme “Cohortes et collections de données biologiques.” The funders had no role in study design, data collection and analysis, decision to publish, or preparation of the manuscript.** | |

*Give information separately for exposed and unexposed groups.

**Note:** An Explanation and Elaboration article discusses each checklist item and gives methodological background and published examples of transparent reporting. The STROBE checklist is best used in conjunction with this article (freely available on the Web sites of PLoS Medicine at http://www.plosmedicine.org/, Annals of Internal Medicine at http://www.annals.org/, and Epidemiology at http://www.epidem.com/). Information on the STROBE Initiative is available at http://www.strobe-statement.org.
