# Supplementary material for: Differential associations of plasma lipids with incident dementia and dementia subtypes in the 3C Study: A longitudinal, population-based prospective cohort study
Source: PLoS Med. 2017 Mar 28;14(3):e1002265. doi: 10.1371/journal.pmed.1002265 (PMC5369688; doi:10.1371/journal.pmed.1002265)
Supplement: S1 Table — (DOCX) [file pmed.1002265.s003.docx]

S1 Table. Correlations between lipid fractions

|  | HDL-C | | LDL-C | | TC | |
| --- | --- | --- | --- | --- | --- | --- |
|  | Correlation coefficient | p | Correlation coefficient | p | Correlation coefficient | p |
| TG | -0.47957 | <0.0001 | 0.20840 | <0.0001 | 0.23740 | <0.0001 |
| HDL-C |  |  | 0.02111 | 0.069 | 0.30698 | <0.0001 |
| LDL-C |  |  |  |  | 0.92823 | <0.0001 |

HDL: high-density lipoprotein cholesterol; LDL: low-density lipoprotein cholesterol; TC: total cholesterol; TG: triglycerides
