# Supplementary material for: Differential associations of plasma lipids with incident dementia and dementia subtypes in the 3C Study: A longitudinal, population-based prospective cohort study
Source: PLoS Med. 2017 Mar 28;14(3):e1002265. doi: 10.1371/journal.pmed.1002265 (PMC5369688; doi:10.1371/journal.pmed.1002265)
Supplement: S2 Table — (DOCX) [file pmed.1002265.s004.docx]

S2 Table. Associations between lipid concentrations at baseline and incident dementia over a 13-year period, using statin intake instead of lipid-lowering drug intake as adjustment

|  | **TG** | | | **HDL-C** | | | **LDL-C** | | | | **TC** | | |
| --- | --- | --- | --- | --- | --- | --- | --- | --- | --- | --- | --- | --- | --- |
|  | **n/N** | **HR (95%CI)** | **p** | **n/N** | **HR (95%CI)** | **p** | | **n/N** | **HR (95%CI)** | **p** | **n/N** | **HR (95%CI)** | **p** |
| ***Model 2b: adjusted for gender, education, center, education*log(age) & vascular risk factors (including statins and not lipid lowering drugs)‡*** | | | | | | | | | | | | | |
| All dementia | 761/7376 | 1.05 (0.96, 1.15) | 0.2502 | 761/7376 | 0.98 (0.90, 1.08) | 0.6928 | | 761/7376 | 1.09 (1.02, 1.18) | 0.0173 | 762/7402 | 1.09 (1.01, 1.18) | 0.0282 |
| Alzheimer’s disease | 522/7376 | 0.99 (0.89, 1.11) | 0.8884 | 522/7376 | 0.97 (0.87, 1.08) | 0.5795 | | 522/7376 | 1.16 (1.06, 1.27) | 0.0012 | 523/7402 | 1.15 (1.05, 1.26) | 0.0034 |
| Mixed or vascular dem. | 150/7376 | 1.18 (0.97, 1.44) | 0.0914 | 150/7376 | 1.06 (0.86, 1.30) | 0.5769 | | 150/7376 | 0.98 (0.83, 1.17) | 0.8593 | 150/7402 | 1.01 (0.85, 1.21) | 0.8857 |
| ***Model 4b: adjusted for gender, education, center, education*log(age), vascular risk factors (including statins and not lipid lowering drugs)‡ & APOEε4 carrier status§*** | | | | | | | | | | | | | |
| All dementia | 755/7344 | 1.07 (0.98, 1.17) | 0.1175 | 755/7344 | 1.00 (0.91, 1.09) | 0.9290 | | 755/7344 | 1.06 (0.98, 1.14) | 0.1277 | 756/7369 | 1.06 (0.98, 1.15) | 0.1325 |
| Alzheimer’s disease | 518/7344 | 1.01 (0.91, 1.13) | 0.8113 | 518/7344 | 0.99 (0.88, 1.10) | 0.8138 | | 518/7344 | 1.12 (1.02, 1.22) | 0.0133 | 519/7369 | 1.12 (1.02, 1.22) | 0.0198 |
| Mixed or vascular dem. | 149/7344 | 1.20 (0.99, 1.46) | 0.0699 | 149/7344 | 1.09 (0.89, 1.34) | 0.4225 | | 149/7344 | 0.96 (0.81, 1.13) | 0.6017 | 149/7369 | 0.99 (0.83, 1.18) | 0.9389 |

APOE: apolipoprotein E; CI: confidence interval; dem.: dementia; HDL-C: high-density lipoprotein cholesterol; HR: hazard ratio; LDL-C: low-density lipoprotein cholesterol; TG: log-transformed triglycerides; TC: total cholesterol; † age represents age at last follow-up or dementia; ‡ vascular risk factors: i) for HDL-C, LDL-C, TG: the three lipid fractions, body mass index, antihypertensive medication, antihypertensive drug*log(age), systolic blood pressure, lipid-lowering medication, smoking status, diabetes, history of cardiovascular disease (for TG, LDL-C, HDL-C) or ii) for TC: TG, body mass index, antihypertensive medication, systolic blood pressure, lipid-lowering medication, smoking status, diabetes, history of cardiovascular disease; Results are given per SD of lipid fraction (TG=0.417; LDL=0.854; HDL=0.401; TC=0.974);
