# Supplementary material for: Differential associations of plasma lipids with incident dementia and dementia subtypes in the 3C Study: A longitudinal, population-based prospective cohort study
Source: PLoS Med. 2017 Mar 28;14(3):e1002265. doi: 10.1371/journal.pmed.1002265 (PMC5369688; doi:10.1371/journal.pmed.1002265)
Supplement: S3 Table — (DOCX) [file pmed.1002265.s005.docx]

S3 Table. Associations between lipid concentrations at baseline and 13-year incident dementia using Cox models accounting for competing risk of death

|  | **TG** | | | **HDL-C** | | | | **LDL-C** | | | | **TC** | | | |
| --- | --- | --- | --- | --- | --- | --- | --- | --- | --- | --- | --- | --- | --- | --- | --- |
|  | n/N | HR (95%CI) | p | n/N | HR (95%CI) | p | n/N | | HR (95%CI) | p | n/N | | HR (95%CI) | p |  |
| ***Model 1: adjusted for sex, education, center, education*log(age)†*** | | | | | | | | | | | | | | | |
| All dementia | 778/7466 | 1.10 (1.03, 1.18) | 0.0060 | 779/7467 | 0.93 (0.86, 1.00) | 0.0487 | 776/7440 | | 1.08 (1.01, 1.16) | 0.0315 | 779/7470 | | 1.08 (1.00, 1.16) | 0.0393 |  |
| Alzheimer’s disease | 531/7466 | 1.06 (0.97, 1.15) | 0.1975 | 532/7467 | 0.95 (0.87, 1.04) | 0.2710 | 529/7440 | | 1.14 (1.05, 1.23) | 0.0024 | 532/7470 | | 1.13 (1.04, 1.23) | 0.0046 |  |
| Mixed or vascular dem. | 154/7466 | 1.21 (1.03, 1.41) | 0.0188 | 154/7467 | 0.90 (0.76, 1.07) | 0.2420 | 154/7440 | | 1.00 (0.85, 1.17) | 0.9590 | 154/7470 | | 1.02 (0.86, 1.19) | 0.8548 |  |
| ***Model 4: adjusted for sex, education, center, education*log(age)†, vascular risk factors‡ & APOEε4 carrier status*** | | | | | | | | | | | | | | | |
| All dementia | 755/7344 | 1.06 (0.97, 1.16) | 0.1829 | 755/7344 | 0.99 (0.90, 1.08) | 0.8287 | 755/7344 | | 1.07 (1.00, 1.16) | 0.0655 | 756/7369 | | 1.07 (0.99, 1.16) | 0.0883 |  |
| Alzheimer’s disease | 518/7344 | 1.01 (0.91, 1.12) | 0.8600 | 518/7344 | 0.98 (0.88, 1.10) | 0.7540 | 518/7344 | | 1.14 (1.04, 1.24) | 0.0055 | 519/7369 | | 1.13 (1.03, 1.24) | 0.0112 |  |
| Mixed or vascular dem. | 149/7344 | 1.15 (0.95, 1.40) | 0.1631 | 149/7344 | 1.07 (0.87, 1.31) | 0.5270 | 149/7344 | | 0.97 (0.81, 1.15) | 0.7050 | 149/7369 | | 1.00 (0.84, 1.19) | 0.9872 |  |

APOE: apolipoprotein E; CI: confidence interval; dem.: dementia; HDL-C: high-density lipoprotein cholesterol; HR: hazard ratio; LDL-C: low-density lipoprotein cholesterol; TG: log-transformed triglycerides; TC: total cholesterol; † age represents age at last follow-up, age at dementia onset or age at death; ‡ vascular risk factors: HDL-C, LDL-C, TG, body mass index, antihypertensive medication, systolic blood pressure, lipid-lowering medication, smoking status, diabetes, history of cardiovascular disease (for TG, LDL-C, HDL-C) or TG, body mass index, antihypertensive medication, systolic blood pressure, lipid-lowering medication, smoking status, diabetes, history of cardiovascular disease (for TC); Results are given per SD of lipid fraction (TG=0.417; LDL=0.854; HDL=0.401; TC=0.974);
