# Supplementary material for: Differential associations of plasma lipids with incident dementia and dementia subtypes in the 3C Study: A longitudinal, population-based prospective cohort study
Source: PLoS Med. 2017 Mar 28;14(3):e1002265. doi: 10.1371/journal.pmed.1002265 (PMC5369688; doi:10.1371/journal.pmed.1002265)
Supplement: S4 Table — (DOCX) [file pmed.1002265.s006.docx]

S4 Table. Association between lipid concentrations at baseline and incident dementia over a 13-year period, stratified by median age.

|  | **TG** | | | | | |  | **HDL-C** | | | | | |  |
| --- | --- | --- | --- | --- | --- | --- | --- | --- | --- | --- | --- | --- | --- | --- |
|  | **Age≤73.15** | | | **Age>73.15** | | | **pi** | **Age≤73.15** | | | **Age>73.15** | | | **pi** |
|  | n/N | HR (95%CI) | p | n/N | HR (95%CI) | p |  | n/N | HR (95%CI) | p | n/N | HR (95%CI) | p |  |
| ***Model 1: adjusted for sex, education, center, education*log(age)†*** | | | | | | | | | | | | | | |
| All dementia | 200/3732 | 1.03 (0.90, 1.17) | 0.7150 | 578/3734 | 1.14 (1.05, 1.23) | 0.0027 | *0.3118* | 200/3732 | 0.89 (0.76, 1.04) | 0.1319 | 579/3735 | 0.94 (0.86, 1.02) | 0.1563 | *0.1315* |
| Alzheimer’s disease | 133/3732 | 0.92 (0.77, 1.09) | 0.3200 | 398/3734 | 1.11 (1.01, 1.23) | 0.0363 | *0.0932* | 133/3732 | 0.93 (0.77, 1.12) | 0.4586 | 399/3735 | 0.96 (0.86, 1.06) | 0.4101 | *0.2235* |
| Mixed or vascular dem. | 31/3732 | 1.17 (0.84, 1.64) | 0.3440 | 123/3734 | 1.23 (1.03, 1.46) | 0.0241 | *0.8450* | 31/3732 | 0.75 (0.50, 1.13) | 0.1744 | 123/3735 | 0.93 (0.77, 1.13) | 0.4702 | *0.3182* |
|  |  |  |  |  |  |  |  |  |  |  |  |  |  |  |
|  | **LDL-C** | | | | | |  | **TC** | | | | | |  |
|  | **Age≤73.15** | | | **Age>73.15** | | | **pi** | **Age≤73.15** | | | **Age>73.15** | | | **pi** |
|  | **n/N** | **HR (95%CI)** | **p** | **n/N** | **HR (95%CI)** | **p** |  | **n/N** | **HR (95%CI)** | **p** | **n/N** | **HR (95%CI)** | **p** |  |
| ***Model 1: adjusted for sex, education, center, education*log(age)†*** | | | | | | | | | | | | | | |
| All dementia | 200/3716 | 0.99 (0.86, 1.14) | 0.8698 | 576/3724 | 1.10 (1.01, 1.19) | 0.0247 | *0.1592* | 200/3734 | 0.95 (0.82, 1.10) | 0.5241 | 579/3736 | 1.11 (1.02, 1.20) | 0.0139 | *0.0313* |
| Alzheimer’s disease | 133/3716 | 1.05 (0.88, 1.24) | 0.5931 | 396/3724 | 1.15 (1.05, 1.27) | 0.0035 | *0.2419* | 133/3734 | 0.99 (0.83, 1.19) | 0.9483 | 399/3736 | 1.16 (1.06, 1.28) | 0.0022 | *0.0468* |
| Mixed or vascular dem. | 31/3716 | 0.87 (0.60, 1.26) | 0.4618 | 123/3724 | 1.02 (0.85, 1.21) | 0.8637 | *0.4794* | 31/3734 | 0.82 (0.56, 1.21) | 0.3218 | 123/3736 | 1.05 (0.88, 1.26) | 0.5729 | *0.2554* |

CI: confidence interval; dem. : dementia ; HDL-C: high-density lipoprotein cholesterol; HR : hazard ratio; LDL-C: low-density lipoprotein cholesterol; pi: p-value for interaction; TC: total cholesterol; TG: log-transformed triglycerides; † age represents age at last follow-up or dementia; Results are given per SD of lipid fraction (TG=0.417; LDL=0.854; HDL=0.401; TC=0.974);
