# Supplementary material for: Differential associations of plasma lipids with incident dementia and dementia subtypes in the 3C Study: A longitudinal, population-based prospective cohort study
Source: PLoS Med. 2017 Mar 28;14(3):e1002265. doi: 10.1371/journal.pmed.1002265 (PMC5369688; doi:10.1371/journal.pmed.1002265)
Supplement: S5 Table — (DOCX) [file pmed.1002265.s007.docx]

S5 Table. Association between lipid concentrations at baseline and incident dementia over a 13-year period, stratified by sex.

|  | **TG** | | | | | |  | **HDL-C** | | | | | |  |
| --- | --- | --- | --- | --- | --- | --- | --- | --- | --- | --- | --- | --- | --- | --- |
|  | **Men** | | | **Women** | | | **pi** | **Men** | | | **Women** | | | **pi** |
|  | n/N | HR (95%CI) | p | n/N | HR (95%CI) | p |  | n/N | HR (95%CI) | p | n/N | HR (95%CI) | p |  |
| ***Model 1: adjusted for education, center, education*log(age)†*** | | | | | | | | | | | | | | |
| All dementia | 268/2910 | 1.10 (0.98, 1.24) | 0.1066 | 510/4556 | 1.11 (1.02, 1.21) | 0.0196 | *0.91* | 269/2911 | 0.95 (0.82, 1.09) | 0.4415 | 510/4556 | 0.91 (0.83, 1.00) | 0.0473 | *0.6452* |
| Alzheimer’s disease | 176/2910 | 1.10 (0.95, 1.26) | 0.2100 | 355/4556 | 1.04 (0.93, 1.16) | 0.4691 | *0.52* | 177/2911 | 0.97 (0.81, 1.15) | 0.6965 | 355/4556 | 0.94 (0.85, 1.05) | 0.2617 | *0.7787* |
| Mixed or vascular dem. | 57/2910 | 1.05 (0.81, 1.36) | 0.6920 | 97/4556 | 1.32 (1.08, 1.61) | 0.0056 | *0.17* | 57/2911 | 0.96 (0.70, 1.32) | 0.8083 | 97/4556 | 0.87 (0.70, 1.07) | 0.1794 | *0.5556* |
|  |  |  |  |  |  |  |  |  |  |  |  |  |  |  |
|  | **LDL-C** | | | | | |  | **TC** | | | | | |  |
|  | **Men** | | | **Women** | | | **pi** | **Men** | | | **Women** | | | **pi** |
|  | **n/N** | **HR (95%CI)** | **p** | **n/N** | **HR (95%CI)** | **p** |  | **n/N** | **HR (95%CI)** | **p** | **n/N** | **HR (95%CI)** | **p** |  |
| ***Model 1: adjusted for education, center, education*log(age)†*** | | | | | | | | | | | | | | |
| All dementia | 267/2891 | 1.03 (0.90, 1.17) | 0.6689 | 509/4549 | 1.09 (1.00, 1.18) | 0.0480 | *0.5034* | 269/2912 | 1.06 (0.94, 1.21) | 0.3398 | 510/4558 | 1.07 (0.98, 1.16) | 0.1199 | *0.9904* |
| Alzheimer’s disease | 175/2891 | 1.10 (0.94, 1.29) | 0.2131 | 354/4549 | 1.13 (1.03, 1.25) | 0.0111 | *0.8057* | 177/2912 | 1.15 (0.98, 1.34) | 0.0772 | 355/4558 | 1.11 (1.00, 1.23) | 0.0452 | *0.6383* |
| Mixed or vascular dem. | 57/2891 | 0.93 (0.70, 1.23) | 0.6017 | 97/4549 | 1.02 (0.84, 1.24) | 0.8492 | *0.6343* | 57/2912 | 0.95 (0.72, 1.26) | 0.7115 | 97/4558 | 1.04 (0.85, 1.26) | 0.7087 | *0.6607* |

CI: confidence interval; dem. : dementia ; HDL-C: high-density lipoprotein cholesterol; HR : hazard ratio; LDL-C: low-density lipoprotein cholesterol; pi: p-value for interaction; TC: total cholesterol; TG: log-transformed triglycerides; † age represents age at last follow-up or dementia; Results are given per SD of lipid fraction (TG=0.417; LDL=0.854; HDL=0.401; TC=0.974);
