# Supplementary material for: Differential associations of plasma lipids with incident dementia and dementia subtypes in the 3C Study: A longitudinal, population-based prospective cohort study
Source: PLoS Med. 2017 Mar 28;14(3):e1002265. doi: 10.1371/journal.pmed.1002265 (PMC5369688; doi:10.1371/journal.pmed.1002265)
Supplement: S6 Table — (DOCX) [file pmed.1002265.s008.docx]

S6 Table. Association between baseline lipid contrations at baseline and incident dementia over a 13-year period, adjusted for lipid-lowering drug intake and stratified by sex

|  | **TG** | | | | | |  | **HDL-C** | | | | | |  |
| --- | --- | --- | --- | --- | --- | --- | --- | --- | --- | --- | --- | --- | --- | --- |
|  | **Men** | | | **Women** | | | **pi** | **Men** | | | **Women** | | | **pi** |
|  | n/N | HR (95%CI) | p | n/N | HR (95%CI) | p |  | n/N | HR (95%CI) | p | n/N | HR (95%CI) | p |  |
| ***Model 1: adjusted for education, center, education*log(age)†*** | | | | | | | | | | | | | | |
| All dementia | 268/2910 | 1.10 (0.98, 1.24) | 0.1002 | 510/4556 | 1.11 (1.02, 1.21) | 0.018 | *0.8993* | 269/2911 | 0.95 (0.82, 1.09) | 0.4473 | 510/4556 | 0.91 (0.83, 1.00) | 0.0511 | *0.6507* |
| Alzheimer’s disease | 176/2910 | 1.10 (0.95, 1.26) | 0.2139 | 355/4556 | 1.04 (0.94, 1.16) | 0.4572 | *0.5310* | 177/2911 | 0.97 (0.81, 1.15) | 0.6877 | 355/4556 | 0.94 (0.85, 1.05) | 0.2683 | *0.7896* |
| Mixed or vascular dem. | 57/2910 | 1.06 (0.82, 1.37) | 0.6583 | 97/4556 | 1.32 (1.08, 1.61) | 0.0057 | *0.1771* | 57/2911 | 0.96 (0.70, 1.32) | 0.8069 | 97/4556 | 0.87 (0.70, 1.07) | 0.1763 | *0.5447* |
|  |  |  |  |  |  |  |  |  |  |  |  |  |  |  |
|  | **LDL-C** | | | | | |  | **TC** | | | | | |  |
|  | **Men** | | | **Women** | | | **pi** | **Men** | | | **Women** | | | **pi** |
|  | **n/N** | **HR (95%CI)** | **p** | **n/N** | **HR (95%CI)** | **p** |  | **n/N** | **HR (95%CI)** | **p** | **n/N** | **HR (95%CI)** | **p** |  |
| ***Model 1: adjusted for education, center, education*log(age)†*** | | | | | | | | | | | | | | |
| All dementia | 267/2891 | 1.02 (0.90, 1.16) | 0.7716 | 509/4549 | 1.11 (1.02, 1.21) | 0.0182 | *0.4837* | 269/2912 | 1.06 (0.93, 1.20) | 0.3861 | 510/4558 | 1.09 (1.00, 1.19) | 0.0591 | *0.9748* |
| Alzheimer’s disease | 175/2891 | 1.11 (0.95, 1.30) | 0.1808 | 354/4549 | 1.15 (1.04, 1.28) | 0.0052 | *0.7683* | 177/2912 | 1.16 (0.99, 1.36) | 0.064 | 355/4558 | 1.12 (1.01, 1.25) | 0.0272 | *0.6869* |
| Mixed or vascular dem. | 57/2891 | 0.91 (0.68, 1.20) | 0.4919 | 97/4549 | 1.01 (0.83, 1.24) | 0.8904 | *0.6549* | 57/2912 | 0.93 (0.70, 1.24) | 0.6202 | 97/4558 | 1.03 (0.84, 1.27) | 0.7422 | *0.6875* |

CI: confidence interval; dem. : dementia ; HDL-C: high-density lipoprotein cholesterol; HR : hazard ratio; LDL-C: low-density lipoprotein cholesterol; pi: p-value for interaction; TC: total cholesterol; TG: log-transformed triglycerides; † age represents age at last follow-up or dementia; Results are given per SD of lipid fraction (TG=0.417; LDL=0.854; HDL=0.401; TC=0.974);
