# Supplementary material for: Differential associations of plasma lipids with incident dementia and dementia subtypes in the 3C Study: A longitudinal, population-based prospective cohort study
Source: PLoS Med. 2017 Mar 28;14(3):e1002265. doi: 10.1371/journal.pmed.1002265 (PMC5369688; doi:10.1371/journal.pmed.1002265)
Supplement: S7 Table — (DOCX) [file pmed.1002265.s009.docx]

S7 Table. Association between lipid concentrations at baseline and incident dementia over a 13-year period, stratified by education.

|  | **TG** | | | | | |  | **HDL-C** | | | | | | |  | | |  |
| --- | --- | --- | --- | --- | --- | --- | --- | --- | --- | --- | --- | --- | --- | --- | --- | --- | --- | --- |
|  | **No education or primary school** | | | **Secondary school or more** | | | **pi** | **No education or primary school** | | | **Secondary school or more** | | | | **pi** | | |  |
|  | n/N | HR (95%CI) | p | n/N | HR (95%CI) | p |  | n/N | HR (95%CI) | p | | n/N | HR (95%CI) | p | |  | | |
| ***Model 1: adjusted for sex, center*** | | | | | | | | | | | | | | | | |  |  |
| All dementia | 316/2301 | 1.10 (0.98, 1.22) | 0.0942 | 462/5165 | 1.13 (1.03, 1.23) | 0.0128 | *0.7711* | 316/2300 | 0.89 (0.79, 1.01) | 0.0620 | | 463/5167 | 0.94 (0.85, 1.04) | 0.2180 | | *0.3556* | | |
| Alzheimer’s disease | 228/2301 | 1.07 (0.94, 1.21) | 0.3232 | 303/5165 | 1.07 (0.95, 1.20) | 0.2597 | *0.9865* | 228/2300 | 0.86 (0.75, 1.00) | 0.0461 | | 304/5167 | 1.00 (0.89, 1.13) | 0.9445 | | *0.0923* | | |
| Mixed or vascular dem. | 60/2301 | 1.19 (0.93, 1.51) | 0.1693 | 94/5165 | 1.24 (1.01, 1.52) | 0.0357 | *0.8213* | 60/2300 | 0.94 (0.71, 1.24) | 0.6590 | | 94/5167 | 0.86 (0.68, 1.07) | 0.1723 | | *0.9344* | | |
|  |  |  |  |  |  |  |  |  |  |  | |  |  |  | |  | | |
|  | **LDL-C** | | | | | |  | **TC** | | | | | | |  | | |  |
|  | **No education or primary school** | | | **Secondary school or more** | | | **pi** | **No education or primary school** | | | **Secondary school or more** | | | | **pi** | | |  |
|  | **n/N** | **HR (95%CI)** | **p** | **n/N** | **HR (95%CI)** | **p** |  | **n/N** | **HR (95%CI)** | **p** | | **n/N** | **HR (95%CI)** | **p** | |  | | |
| ***Model 1: adjusted for sex, center*** | | | | | | | | | | | | | | | | |  |  |
| All dementia | 315/2292 | 1.10 (0.99, 1.22) | 0.0878 | 461/5148 | 1.05 (0.96, 1.15) | 0.2608 | *0.6281* | 316/2301 | 1.07 (0.96, 1.19) | 0.2381 | | 463/5169 | 1.07 (0.98, 1.17) | 0.1380 | | *0.8361* | | |
| Alzheimer’s disease | 227/2292 | 1.16 (1.02, 1.31) | 0.0217 | 302/5148 | 1.10 (0.99, 1.23) | 0.0778 | *0.6292* | 228/2301 | 1.10 (0.97, 1.26) | 0.1374 | | 304/5169 | 1.13 (1.01, 1.27) | 0.0262 | | *0.6718* | | |
| Mixed or vascular dem. | 60/2292 | 0.94 (0.73, 1.21) | 0.6278 | 94/5148 | 1.02 (0.84, 1.25) | 0.8232 | *0.4941* | 60/2301 | 0.97 (0.74, 1.25) | 0.7903 | | 94/5169 | 1.03 (0.84, 1.27) | 0.7443 | | *0.4835* | | |

CI: confidence interval; dem. : dementia ; HDL-C: high-density lipoprotein cholesterol; HR : hazard ratio; LDL-C: low-density lipoprotein cholesterol; pi: p-value for interaction; TC: total cholesterol; TG: log-transformed triglycerides; Results are given per SD of lipid fraction (TG=0.417; LDL=0.854; HDL=0.401; TC=0.974);
