# Supplementary material for: Differential associations of plasma lipids with incident dementia and dementia subtypes in the 3C Study: A longitudinal, population-based prospective cohort study
Source: PLoS Med. 2017 Mar 28;14(3):e1002265. doi: 10.1371/journal.pmed.1002265 (PMC5369688; doi:10.1371/journal.pmed.1002265)
Supplement: S8 Table — (DOCX) [file pmed.1002265.s010.docx]

S8 Table. Association between lipid concentrations at baseline and incident dementia over a 13-year period, stratified by *APOEε*4 carrier status.

|  | **TG** | | | | | |  | **HDL-C** | | | | | |  |
| --- | --- | --- | --- | --- | --- | --- | --- | --- | --- | --- | --- | --- | --- | --- |
|  | **APOEε4 noncarrier** | | | **APOEε4 carrier** | | | **pi** | **APOEε4 noncarrier** | | | **APOEε4 carrier** | | | **pi** |
|  | n/N | HR (95%CI) | p | n/N | HR (95%CI) | p |  | n/N | HR (95%CI) | p | n/N | HR (95%CI) | p |  |
| ***Model 1: adjusted for sex, education, center, education*log(age)†*** | | | | | | | | | | | | | |  |
| All dementia | 556/5940 | 1.11 (1.02, 1.20) | 0.0183 | 214/1489 | 1.11 (0.98, 1.27) | 0.1100 | *0.9384* | 557/5941 | 0.94 (0.86, 1.03) | 0.1713 | 214/1489 | 0.91 (0.78, 1.05) | 0.2056 | *0.6697* |
| Alzheimer’s disease | 373/5940 | 1.05 (0.94, 1.16) | 0.3941 | 153/1489 | 1.10 (0.94, 1.29) | 0.2144 | *0.6013* | 374/5941 | 0.98 (0.88, 1.09) | 0.6786 | 153/1489 | 0.90 (0.75, 1.07) | 0.2309 | *0.4030* |
| Mixed or vascular dem | 110/5940 | 1.24 (1.03, 1.49) | 0.0237 | 42/1489 | 1.09 (0.80, 1.47) | 0.5938 | *0.4860* | 110/5941 | 0.88 (0.72, 1.08) | 0.2253 | 42/1489 | 1.04 (0.75, 1.44) | 0.8241 | *0.4036* |
|  |  |  |  |  |  |  |  |  |  |  |  |  |  |  |
|  | **LDL-C** | | | | | |  | **TC** | | | | | |  |
|  | **APOEε4 noncarrier** | | | **APOEε4 carrier** | | | **pi** | **APOEε4 noncarrier** | | | **APOEε4 carrier** | | | **pi** |
|  | **n/N** | **HR (95%CI)** | **p** | **n/N** | **HR (95%CI)** | **p** |  | **n/N** | **HR (95%CI)** | **p** | **n/N** | **HR (95%CI)** | **p** |  |
| ***Model 1: adjusted for sex, education, center, education*log(age)†*** | | | | | | | | | | | | | | |
| All dementia | 555/5921 | 1.04 (0.95, 1.13) | 0.3841 | 213/1483 | 1.06 (0.94, 1.20) | 0.3314 | *0.7548* | 557/5944 | 1.05 (0.96, 1.14) | 0.2724 | 214/1489 | 1.05 (0.93, 1.20) | 0.4193 | *0.9646* |
| Alzheimer’s disease | 372/5921 | 1.08 (0.98, 1.20) | 0.1288 | 152/1483 | 1.13 (0.98, 1.30) | 0.1035 | *0.6782* | 374/5944 | 1.09 (0.98, 1.21) | 0.1003 | 153/1489 | 1.11 (0.96, 1.29) | 0.1569 | *0.8692* |
| Mixed or vascular dem | 110/5921 | 1.01 (0.83, 1.22) | 0.9153 | 42/1483 | 0.88 (0.65, 1.18) | 0.3900 | *0.5143* | 110/5944 | 1.04 (0.85, 1.26) | 0.7165 | 42/1489 | 0.90 (0.66, 1.22) | 0.5056 | *0.5490* |

CI: confidence interval; dem. : dementia ; HDL-C: high-density lipoprotein cholesterol; HR : hazard ratio; LDL-C: low-density lipoprotein cholesterol; pi: p-value for interaction; TC: total cholesterol; TG: log-transformed triglycerides; † age represents age at last follow-up or dementia; Results are given per SD of lipid fraction (TG=0.417; LDL=0.854; HDL=0.401; TC=0.974);
