# Supplementary material for: Differential associations of plasma lipids with incident dementia and dementia subtypes in the 3C Study: A longitudinal, population-based prospective cohort study
Source: PLoS Med. 2017 Mar 28;14(3):e1002265. doi: 10.1371/journal.pmed.1002265 (PMC5369688; doi:10.1371/journal.pmed.1002265)
Supplement: S9 Table — (DOCX) [file pmed.1002265.s011.docx]

S9 Table. Association between lipid concentrations at baseline and incident dementia over a 13-year period, stratified by lipid-lowering drug intake.

|  | **TG** | | | | | |  | **HDL-C** | | | | | |  |
| --- | --- | --- | --- | --- | --- | --- | --- | --- | --- | --- | --- | --- | --- | --- |
|  | **No lipid lowering drugs** | | | **Lipid lowering drugs** | | | **pi** | **No lipid lowering drugs** | | | **Lipid lowering drugs** | | | **pi** |
|  | n/N | HR (95%CI) | p | n/N | HR (95%CI) | p |  | n/N | HR (95%CI) | p | n/N | HR (95%CI) | p |  |
| ***Model 1: adjusted for sex, education, center, education*log(age)†*** | | | | | | | | | | | | | | |
| All dementia | 546/5169 | 1.11 (1.02, 1.21) | 0.0197 | 232/2297 | 1.10 (0.97, 1.24) | 0.1254 | *0.8193* | 546/5169 | 0.93 (0.85, 1.02) | 0.1124 | 233/2298 | 0.93 (0.81, 1.06) | 0.2882 | *0.7503* |
| Alzheimer’s disease | 369/5169 | 1.08 (0.97, 1.20) | 0.1525 | 162/2297 | 1.02 (0.88, 1.18) | 0.8133 | *0.4767* | 369/5169 | 0.94 (0.84, 1.05) | 0.2970 | 163/2298 | 0.98 (0.83, 1.15) | 0.7791 | *0.7604* |
| Mixed or vascular dem | 114/5169 | 1.19 (0.99, 1.44) | 0.0709 | 40/2297 | 1.25 (0.95, 1.64) | 0.1161 | *0.7124* | 114/5169 | 0.90 (0.74, 1.10) | 0.3094 | 40/2298 | 0.86 (0.62, 1.21) | 0.3946 | *0.9517* |
|  |  |  |  |  |  |  |  |  |  |  |  |  |  |  |
|  | **LDL-C** | | | | | |  | **TC** | | | | | |  |
|  | **No lipid lowering drugs** | | | **Lipid lowering drugs** | | | **pi** | **No lipid lowering drugs** | | | **Lipid lowering drugs** | | | **pi** |
|  | **n/N** | **HR (95%CI)** | **p** | **n/N** | **HR (95%CI)** | **p** |  | **n/N** | **HR (95%CI)** | **p** | **n/N** | **HR (95%CI)** | **p** |  |
| ***Model 1: adjusted for sex, education, center, education*log(age)†*** | | | | | | | | | | | | | | |
| All dementia | 544/5151 | 1.08 (1.00, 1.18) | 0.0623 | 232/2289 | 1.09 (0.95, 1.26) | 0.2049 | *0.7717* | 546/5172 | 1.08 (0.99, 1.18) | 0.0688 | 233/2298 | 1.09 (0.95, 1.25) | 0.2192 | *0.6922* |
| Alzheimer’s disease | 367/5151 | 1.13 (1.03, 1.25) | 0.0133 | 162/2289 | 1.18 (1.00, 1.38) | 0.0466 | *0.6170* | 369/5172 | 1.13 (1.02, 1.25) | 0.0187 | 163/2298 | 1.17 (0.99, 1.37) | 0.0651 | *0.6778* |
| Mixed or vascular dem | 114/5151 | 0.99 (0.82, 1.19) | 0.9080 | 40/2289 | 0.96 (0.68, 1.37) | 0.8319 | *0.8855* | 114/5172 | 1.00 (0.83, 1.21) | 0.9737 | 40/2298 | 0.99 (0.70, 1.41) | 0.9675 | *0.9627* |

CI: confidence interval; dem. : dementia ; HDL-C: high-density lipoprotein cholesterol; HR : hazard ratio; LDL-C: low-density lipoprotein cholesterol; pi: p-value for interaction; TC: total cholesterol; TG: log-transformed triglycerides; † age represents age at last follow-up or dementia; Results are given per SD of lipid fraction (TG=0.417; LDL=0.854; HDL=0.401; TC=0.974);
