# Supplementary material for: Differential associations of plasma lipids with incident dementia and dementia subtypes in the 3C Study: A longitudinal, population-based prospective cohort study
Source: PLoS Med. 2017 Mar 28;14(3):e1002265. doi: 10.1371/journal.pmed.1002265 (PMC5369688; doi:10.1371/journal.pmed.1002265)
Supplement: S10 Table — (DOCX) [file pmed.1002265.s012.docx]

S10 Table. Associations between lipid concentrations at baseline and incident dementia over a 13-year period, stratified on a variable combining lipid-lowering drug intake and baseline total cholesterol concentration

|  | **No lipid lowering drugs** | | | **Lipid lowering drugs use** | | | | | | | **pi** | **No lipid lowering drugs** | | | **Lipid lowering drugs use** | | | | | | **pi** |
| --- | --- | --- | --- | --- | --- | --- | --- | --- | --- | --- | --- | --- | --- | --- | --- | --- | --- | --- | --- | --- | --- |
|  |  |  |  | **Low TC (<6.2 mmol/L)** | | | **High TC ( ≥ 6.2 mmol/L)** | | |  | |  | | | **Low TC (<6.2 mmol/L)** | | | **High TC ( ≥ 6.2 mmol/L)** | | |  |
|  | n/N | HR (95%CI) | p | n/N | HR (95%CI) | p | n/N | HR (95%CI) | p |  | | n/N | HR (95%CI) | p | n/N | HR (95%CI) | p | n/N | HR (95%CI) | p |  |
| ***Model adjusted for gender, education, center, education*log(age)†*** | | | | | | | | | | | | | | | | | | | | |  |
| All dementia | 546/5169 | 1.11 (1.02, 1.21) | 0.0197 | 176/1814 | 1.05 (0.91, 1.22) | 0.4706 | 56/483 | 1.19 (0.94, 1.5) | 0.1501 | *0.6984* | | 546/5169 | 0.93 (0.85, 1.02) | 0.1124 | 176/1814 | 0.95 (0.80, 1.12) | 0.5205 | 57/484 | 0.87 (0.67, 1.12) | 0.2710 | *0.7775* |
| Alzheimer’s disease | 369/5169 | 1.08 (0.97, 1.20) | 0.1525 | 120/1814 | 0.96 (0.8, 1.14) | 0.6325 | 42/483 | 1.10 (0.84, 1.44) | 0.5012 | *0.5068* | | 369/5169 | 0.94 (0.84, 1.05) | 0.2970 | 120/1814 | 1.06 (0.87, 1.29) | 0.5429 | 43/484 | 0.78 (0.57, 1.06) | 0.1168 | *0.2775* |
| Mixed or vascular dem. | 114/5169 | 1.19 (0.99, 1.44) | 0.0709 | 31/1814 | 1.20 (0.85, 1.68) | 0.3039 | 9/483 | 1.53 (0.86, 2.74) | 0.1492 | 0.6457 | | 114/5169 | 0.90 (0.74, 1.10) | 0.3094 | 31/1814 | 0.84 (0.57, 1.26) | 0.4069 | 9/484 | 0.88 (0.45, 1.75) | 0.7200 | 0.9762 |
|  |  |  |  |  |  |  |  |  |  |  | |  |  |  |  |  |  |  |  |  |  |
|  | **LDL-C** | | | | | | | | |  | | **TC** | | | | | | | | |  |
|  | **No lipid lowering drugs** | | | **Lipid lowering drugs use** | | | | | | | **pi** | **No lipid lowering drugs** | | | **Lipid lowering drugs use** | | | | | | **pi** |
|  |  |  |  | **Low TC (<6.2 mmol/L)** | | | **High TC ( ≥ 6.2 mmol/L)** | | |  | |  | | | **Low TC (<6.2 mmol/L)** | | | **High TC ( ≥ 6.2 mmol/L)** | | |  |
|  | **n/N** | **HR (95%CI)** | **p** | **n/N** | **HR (95%CI)** | **p** | **n/N** | **HR (95%CI)** | **p** |  | | **n/N** | **HR (95%CI)** | **p** | **n/N** | **HR (95%CI)** | **p** | **n/N** | **HR (95%CI)** | **p** |  |
| ***Model adjusted for gender, education, center, education*log(age)†*** | | | | | | | | | | | | | | | | | | | | | |
| All dementia | 544/5151 | 1.08 (1.00, 1.18) | 0.0623 | 176/1810 | 0.95 (0.75, 1.20) | 0.6504 | 56/479 | 1.39 (1.01, 1.92) | 0.0446 | *0.1082* | | 546/5172 | 1.08 (0.99, 1.18) | 0.0688 | 176/1814 | 0.94 (0.74, 1.20) | 0.6401 | 57/484 | 1.49 (1.04, 2.14) | 0.0312 | *0.1204* |
| Alzheimer’s disease | 367/5151 | 1.13 (1.03, 1.25) | 0.0133 | 120/1810 | 0.96 (0.72, 1.27) | 0.7541 | 42/479 | 1.64 (1.16, 2.32) | 0.0051 | *0.0294* | | 369/5172 | 1.13 (1.02, 1.25) | 0.0187 | 120/1814 | 1.00 (0.74, 1.34) | 0.9738 | 43/484 | 1.57 (1.05, 2.34) | 0.0290 | *0.1661* |
| Mixed or vascular dem. | 114/5151 | 0.99 (0.82, 1.19) | 0.908 | 31/1810 | 0.91 (0.52, 1.57) | 0.7233 | 9/479 | 0.68 (0.24, 1.95) | 0.4731 | *0.7983* | | 114/5172 | 1.00 (0.83, 1.21) | 0.9737 | 31/1814 | 0.89 (0.51, 1.56) | 0.6785 | 9/484 | 0.96 (0.29, 3.18) | 0.9492 | *0.9607* |

CI: confidence interval; dem. : dementia ; HDL-C: high-density lipoprotein cholesterol; HR : hazard ratio; LDL-C: low-density lipoprotein cholesterol; pi: p-value for interaction; TC: total cholesterol; TG: log-transformed triglycerides; † age represents age at last follow-up or dementia; Results are given per SD of lipid fraction (TG=0.417; LDL=0.854; HDL=0.401; TC=0.974);
