# Supplementary material for: Differential associations of plasma lipids with incident dementia and dementia subtypes in the 3C Study: A longitudinal, population-based prospective cohort study
Source: PLoS Med. 2017 Mar 28;14(3):e1002265. doi: 10.1371/journal.pmed.1002265 (PMC5369688; doi:10.1371/journal.pmed.1002265)
Supplement: S11 Table — (DOCX) [file pmed.1002265.s013.docx]

S11 Table. Associations between lipid concentrations in sex-specific quartiles and 13-year incident dementia

|  | **n/N** | **1^st^ quartile** | **2^nd^ quartile** | | **3^rd^ quartile** | | | **4^th^ quartile** | | | **P for trend** |
| --- | --- | --- | --- | --- | --- | --- | --- | --- | --- | --- | --- |
|  |  | **(4^th^  for HDL-C)** | **(3^rd^ for HDL-C)** | | **(2^nd^ for HDL-C)** | | | **(1^st^ for HDL-C)** | | |  |
|  |  |  | **HR(95%CI)** | **p** | | **HR(95%CI)** | **p** | | **HR(95%CI)** | **p** |  |
| ***Model adjusted for sex, education, center, education*log(age)†*** | | | | | | | | | | | |
| **TG** |  |  |  |  | |  |  | |  |  |  |
| All dementia | 778/7466 | Ref. | 1.06 (0.86, 1.30) | 0.6053 | | 1.14 (0.93, 1.40) | 0.2037 | | 1.33 (1.09, 1.63) | 0.0051 | 0.0032 |
| Alzheimer’s disease | 531/7466 | Ref. | 1.12 (0.87, 1.44) | 0.3753 | | 1.20 (0.93, 1.53) | 0.1538 | | 1.23 (0.96, 1.58) | 0.0999 | 0.0855 |
| Mixed or vascular dem. | 154/7466 | Ref. | 0.85 (0.52, 1.37) | 0.4972 | | 1.10 (0.70, 1.74) | 0.6698 | | 1.35 (0.87, 2.09) | 0.1788 | 0.0921 |
| **HDL-C** |  |  |  |  | |  |  | |  |  |  |
| All dementia | 779/7467 | Ref. | 1.23 (1.00, 1.51) | 0.0476 | | 1.17 (0.95, 1.44) | 0.1450 | | 1.29 (1.06, 1.58) | 0.0130 | 0.0298 |
| Alzheimer’s disease | 532/7467 | Ref. | 1.16 (0.91, 1.49) | 0.2272 | | 1.12 (0.88, 1.44) | 0.3582 | | 1.20 (0.94, 1.52) | 0.1467 | 0.2025 |
| Mixed or vascular dem. | 154/7467 | Ref. | 1.38 (0.87, 2.21) | 0.1729 | | 1.29 (0.81, 2.08) | 0.2836 | | 1.36 (0.85, 2.16) | 0.1977 | 0.2735 |
| **LDL-C** | | | | | | | | | | | |
| All dementia | 776/7440 | Ref. | 1.07 (0.88, 1.31) | 0.4963 | | 0.91 (0.74, 1.12) | 0.3864 | | 1.22 (1.00, 1.48) | 0.0479 | 0.1479 |
| Alzheimer’s disease | 529/7440 | Ref. | 1.16 (0.91, 1.49) | 0.2304 | | 0.97 (0.75, 1.25) | 0.8048 | | 1.36 (1.07, 1.73) | 0.0108 | 0.0420 |
| Mixed or vascular dem. | 154/7440 | Ref. | 0.84 (0.53, 1.31) | 0.4328 | | 0.85 (0.54, 1.32) | 0.4641 | | 0.99 (0.64, 1.52) | 0.9600 | 0.9693 |
| **Total cholesterol** | | | | | | | | | | | |
| All dementia | 779/7470 | Ref. | 1.06 (0.86, 1.29) | 0.6052 | | 1.02 (0.83, 1.24) | 0.8844 | | 1.21 (1.00, 1.48) | 0.0529 | 0.0813 |
| Alzheimer’s disease | 532/7470 | Ref. | 1.17 (0.91, 1.51) | 0.2249 | | 1.22 (0.95, 1.56) | 0.1196 | | 1.36 (1.06, 1.73) | 0.0145 | 0.0154 |
| Mixed or vascular dem. | 154/7470 | Ref. | 0.96 (0.62, 1.49) | 0.8479 | | 0.78 (0.49, 1.24) | 0.2948 | | 1.09 (0.71, 1.67) | 0.7073 | 0.9207 |

CI: confidence interval; dem.: dementia; HDL-C: high-density lipoprotein cholesterol; HR: hazard ratio; LDL-C: low-density lipoprotein cholesterol; MMSE: mini-mental state examination; Ref: reference; TG: triglycerides; † age represents age at last follow-up or dementia;

Quartiles were defined as follows (mmol/L):

TG:

for women: 1^st^ quartile <0.84; 2^nd^ quartile [0.85; 1.07]; 3^rd^ quartile [1.08; 1.43]; 4^th^ quartile ≥1.44

for men: 1^st^ quartile <0.88; 2^nd^ quartile [0.89; 1.15]; 3^rd^ quartile [1.15; 1.54]; 4^th^ quartile ≥1.55

HDL:

for women: 1^st^ quartile ≤1.46; 2^nd^ quartile [1.47; 1.69]; 3^rd^ quartile [1.6900001; 1.97]; 4^th^ quartile ≥1.98;

for men: 1^st^ quartile ≤1.20; 2^nd^ quartile [1.21; 1.40]; 3^rd^ quartile [1.40; 1.63]; 4^th^ quartile ≥1.64

LDL-C:

For women: 1^st^ quartile <3.09; 2^nd^ quartile [3.10-3.64]; 3^rd^ quartile [3.65; 4.24]; 4^th^ quartile ≥4.25

For men: 1^st^ quartile <2.96; 2^nd^ quartile [2.97; 3.49]; 3^rd^ quartile [3.50; 3.99]; 4^th^ quartile ≥4.00

Total cholesterol :

For women: 1^st^ quartile <5.33; 2^nd^ quartile [5.34; 5.93], 3^rd^ quartile [5.94; 6.59], 4^th^ quartile ≥6.60

For men: 1^st^ quartile<4.93; 2^nd^ quartile [4.94; 5.51]; 3^rd^ quartile [5.52; 6.10]; 4^th^ quartile ≥6.10
