# Supplementary material for: Differential associations of plasma lipids with incident dementia and dementia subtypes in the 3C Study: A longitudinal, population-based prospective cohort study
Source: PLoS Med. 2017 Mar 28;14(3):e1002265. doi: 10.1371/journal.pmed.1002265 (PMC5369688; doi:10.1371/journal.pmed.1002265)
Supplement: S1 Text — (DOCX) [file pmed.1002265.s014.docx]

**Differential associations of plasma lipids with incident dementia and subtypes in the 3C-Study: a longitudinal population-based prospective cohort study**

**ANALYSIS PLAN**

The relationship of baseline lipid levels with 13-year incident dementia, will be studied using Cox models, with delayed entry using age as a time scale to avoid the non-proportionality of dementia risk with age.([1](#_ENREF_1)) Data will be censored at the date of dementia diagnosis for cases (median of the interval of the last follow-up visit without dementia and the first follow-up visit with dementia) or at the date of last follow-up for controls. In analyses on dementia subtypes, the subtypes that are not of primary interest will be excluded from analyses.

*Note: Following the reviewers’ comments, we have corrected the analyses of dementia subtypes, censoring alternative dementia subtypes at the time of diagnosis instead of excluding them from the analyses.*

Prerequisite

We will verify the proportional hazard assumption using proportionality tests that assess the statistical significance of interaction terms between time (age at last follow-up or dementia occurrence) and the variables in the model. If this assumption is not verified for some covariates, the relevant Cox models will also include an interaction term for these covariates with time.

Main analyses

The following models will be developed, in each of which one or several interaction terms between time and a covariate might be added, depending on proportional hazard assumption verification:

-Model 1 will be adjusted for study center, gender and educational level.

-To account for potential confounding effect of other vascular risk factors, model 2 will be additionally adjusted for the following vascular risk factors: body mass index (BMI), systolic blood pressure, anti-hypertensive drug intake, smoking status, diabetes, lipid-lowering drug intake and other lipid levels, history of cardiovascular disease.

- To account for potential confounding effect of the *APOE*ε4 carrier-status, model 3 will be based on model 1 and additionally adjusted for *APOE*ε4 genotype. *APOEε4* is a major known risk factor for dementia and Alzheimer’s disease, and it is also a key player in lipid metabolism.([2-4](#_ENREF_2)) Analyses including *APOEε4* carrier-status will be run after exclusion of participants with the *APOEε24* genotype since the *APOEε2* allele is protective factor for Alzheimer’s disease and may “diminish” the effect of *APOEε4*.([2-5](#_ENREF_2))

*Note: Following the reviewers’ comments, we have subsequently replaced model 3 by a model including both additional adjustment for vascular risk factors and APOEε4 genotype. In addition, also following the reviewers’ comments, we are no longer excluding participants with the APOEε24, as this strategy is controversial and leads to a reduction in sample size.*

Notes concerning lipid fractions

Lipid levels will be studied as continuous variables with results reported by standard deviation increase. TG levels will be log-transformed for analyses to remove skewness.

Correction for multiple testing

We will correct for multiple testing using a previously described method,([6](#_ENREF_6)) accounting for the correlation between the different lipid phenotypes examined, and correcting for the estimated number of independent phenotypes.

Secondary analyses

We will first account for competing risk of death by running sensitivity analyses censoring non-demented individuals at the date of last follow-up or date of death (instead of date of last follow-up only as in the primary analyses). Second, we will also perform Cox models using time-on-study as the time scale and generate cumulative incidence graphs, adjusting for age, gender and education, stratified on lipid levels (top quartile [or bottom for HDL-C], vs the rest).

Third, we will examine the robustness of our results by stratifying on *APOE*ε4 carrier status, lipid-lowering drug intake, gender, median age, and educational level (using a dichotomized variable coding no education and primary school vs the rest) and formally test for interaction with these variables.

Fourth, we will explore the relationship between gender-specific lipid quartiles and dementia risk. For significant associations (found in main analyses), linearity will be assessed using a method based on Cox models with delayed entry. The hazard ratio of each class of the lipid fraction divided in 4 classes, in each of which lipid levels is replaced by the value of the mean of the corresponding quartile minus that of the first quartile, will be compared to the confidence interval of the hazard ratio of the corresponding quartile. If the hazard ratio is included in the confidence interval of the corresponding quartile, linearity is respected. We will also use plots of cumulative Martingale residuals against the continuous variable of interest (lipid fractions) to confirm linearity.

1. Thiebaut AC, Benichou J. Choice of time-scale in Cox's model analysis of epidemiologic cohort data: a simulation study. Stat Med. 2004 Dec 30;23(24):3803-20.

2. Farrer LA, Cupples LA, Haines JL, Hyman B, Kukull WA, Mayeux R, et al. Effects of age, sex, and ethnicity on the association between apolipoprotein E genotype and Alzheimer disease. A meta-analysis. APOE and Alzheimer Disease Meta Analysis Consortium. JAMA. 1997 Oct 22-29;278(16):1349-56.

3. Verghese PB, Castellano JM, Holtzman DM. Apolipoprotein E in Alzheimer's disease and other neurological disorders. Lancet Neurol. 2011 Mar;10(3):241-52.

4. Henderson AS, Easteal S, Jorm AF, Mackinnon AJ, Korten AE, Christensen H, et al. Apolipoprotein E allele epsilon 4, dementia, and cognitive decline in a population sample. Lancet. 1995 Nov 25;346(8987):1387-90.

5. Corder EH, Saunders AM, Risch NJ, Strittmatter WJ, Schmechel DE, Gaskell PC, Jr., et al. Protective effect of apolipoprotein E type 2 allele for late onset Alzheimer disease. Nat Genet. 1994 Jun;7(2):180-4.

6. Li J, Ji L. Adjusting multiple testing in multilocus analyses using the eigenvalues of a correlation matrix. Heredity (Edinb). 2005 Sep;95(3):221-7.
